# Electronic Asymmetry Engineering of Fe–N–C Electrocatalyst via Adjacent Carbon Vacancy for Boosting Oxygen Reduction Reaction

**DOI:** 10.1002/advs.202305194

**Published:** 2023-09-26

**Authors:** Huanlu Tu, Haixia Zhang, Yanhui Song, Peizhi Liu, Ying Hou, Bingshe Xu, Ting Liao, Junjie Guo, Ziqi Sun

**Affiliations:** ^1^ Key Laboratory of Interface Science and Engineering in Advanced Materials, Ministry of Education Taiyuan University of Technology Taiyuan 030024 P. R. China; ^2^ School of Chemistry and Physics Queensland University of Technology Brisbane QLD 4001 Australia; ^3^ Materials Institute of Atomic and Molecular Science Shaanxi University of Science and Technology Xi'an 710021 P. R. China; ^4^ School of Mechanical, Medical and Process Engineering Queensland University of Technology Brisbane QLD 4000 Australia; ^5^ Centre for Materials Science Queensland University of Technology Brisbane QLD 4001 Australia

**Keywords:** electronic asymmetry engineering, Fe–N–C catalysts, oxygen reduction reaction, universal pH range

## Abstract

Single‐atomic transition metal–nitrogen–carbon (M–N–C) structures are promising alternatives toward noble‐metal‐based catalysts for oxygen reduction reaction (ORR) catalysis involved in sustainable energy devices. The symmetrical electronic density distribution of the M─N_4_ moieties, however, leads to unfavorable intermediate adsorption and sluggish kinetics. Herein, a Fe–N–C catalyst with electronic asymmetry induced by one nearest carbon vacancy adjacent to Fe─N_4_ is conceptually produced, which induces an optimized d‐band center, lowered free energy barrier, and thus superior ORR activity with a half‐wave potential (*E*_1/2_) of 0.934 V in a challenging acidic solution and 0.901 V in an alkaline solution. When assembled as the cathode of a Zinc–air battery (ZAB), a peak power density of 218 mW cm^−2^ and long‐term durability up to 200 h are recorded, 1.5 times higher than the noble metal‐based Pt/C+RuO_2_ catalyst. This work provides a new strategy on developing efficient M–N–C catalysts and offers an opportunity for the real‐world application of fuel cells and metal–air batteries.

## Graphical abstract

This work provides a novel approach to break the symmetry of the Fe─N_4_ moieties in Fe–N–C catalysts via vacancy engineering. The electronic distribution asymmetry of the Fe–N–C catalysts presents boosting activity and prominent durability in a universal pH range. This facile and cost‐effective approach with scalable production potential demonstrates promising applications in real‐world fuel cells and metal–air batteries.

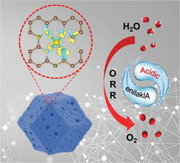

## 1. Introduction

The oxygen reduction reaction (ORR) is a key cathode reaction in clean energy conversion technologies, such as fuel cells and metal–air batteries, which has to be promoted by catalysts, due to its sluggish kinetics.^[^ ^1^, ^2^, ^3^, ^4^ ^]^ It has hitherto been recognized that platinum group metal (PGM) catalysts show the highest ORR performance and are thus considered as the benchmark catalysts.^[^ ^5^, ^6^, ^7^, ^8^, ^9^ ^]^ However, the prohibitive cost, limited resources, and low cooking tolerance of PGM have driven the exploration of high‐efficient noble metal‐free electrocatalysts.^[^ ^10^, ^11^, ^12^ ^]^ As promising alternatives, atomically dispersed single‐atom catalysts (SACs) with excellent catalytic performance have demonstrated great promising in heterogeneous catalysis. Among them, transition metal–nitrogen–carbon (M–N–C) catalysts have received specific attention, due to their outstanding stability by immobilizing the single‐atom active sites by coordinated nitrogen, highly accessible surface areas contributed by 2D carbon material, excellent conductivity of carbon support, flexible choices of single metal atoms, and low materials and fabrication cost.^[^ ^13^, ^14^, ^15^, ^16^, ^17^ ^]^ Particularly, the Fe–N–C catalysts with Fe coordinated with N in the form of Fe─N_4_ configuration possess a Pt‐like behavior for O_2_ adsorption and subsequent O═O bond breaking during the ORR catalysis,^[^ ^18^, ^19^, ^20^ ^]^ which make the Fe–N–C catalysts more promising if we can achieve a high loading level of Fe─N_4_ active sites, maintain the catalytical activity in a universal pH condition, and further improve the catalytic performance via proper electronic structure modulation.^[^ ^21^, ^22^, ^23^ ^]^

However, the Fe‐N_4_ moieties with high symmetrical electronic density distribution are unfavorable for the adsorption of intermediates, thus leading to sluggish kinetic activity.^[^ ^24^, ^25^, ^26^, ^27^ ^]^ Efforts on breaking the structural or electronic symmetry of the Fe─N_4_ moieties, therefore, have been made via heteroatomic substitution of one N atoms by S, P, and B to boost the electrocatalytic activity of the Fe–N–C catalysts.^[^ ^28^, ^29^, ^30^, ^31^, ^32^ ^]^ The doping induced electronic asymmetry facilitates the adsorption behavior of the intermediates and lowers the barrier of the catalytic reaction steps, and thus significantly increases the catalytic activity. The homogeneous doping of non‐metallic elements into the desired Fe─N_4_ moieties, unfortunately, is very challenging. Therefore, further strategies capable of homogenously breaking the electronic symmetry while maintaining good environmental durability are urgently needed.

Herein, an atomically dispersed Fe–N–C catalyst with generated nearest carbon defects adjacent to the F─N_4_ moieties (Fe─N_4_‐Vc) was fabricated from a Fe modified zeolitic imidazolate framework (ZIF‐8), where the Zn atoms were partially replaced by Fe atoms. The Zn evaporation from ZIF‐8 during the thermal activation leaves behind a defect‐rich and N‐rich hierarchical carbon framework, which are ideal host for immobilizing extremely unstable single metal sites to the prominent N ligands and porosities.^[^ ^33^, ^34^, ^35^ ^]^ The generation of carbon vacancy adjacent to the Fe─N_4_ active centers by urea etching led to the redistribution of electronic density of the Fe─N_4_ structure and resulted in an asymmetric electronic state of the new Fe─N_4_‐Vc active sites. Most importantly, the creation of adjacent carbon vacancies is more energy favorable than the heterogeneous non‐metal atoms doping and could be more stable during catalytic applications. Via the carbon vacancy induced asymmetry, the Fe─N_4_‐Vc structure reached a half‐wave potential (*E*_1/2_) of 0.934 V in acidic media and 0.901 V in alkaline media and excellent environmental durability. When assembled as the air cathode, the Zn–air battery (ZAB) achieved a maximum power density of 218 mW cm^−2^ and stable cycling at 10 mA cm^−2^ for >200 h, which much outperform the Pt/C+RuO_2_ benchmark catalysts (149 mW cm^−2^ and 75 h, respectively, tested under the same conditions). This electronic asymmetry engineered Fe─N_4_‐Vc catalyst provides not only enhanced catalytic activity and improved device performance, but also offers a novel approach for designing high‐performance catalysts toward practical energy devices.

## 1 Results and Discussion

### 1.1 Catalyst Synthesis and Structure Characterizations

**Figure 1:**
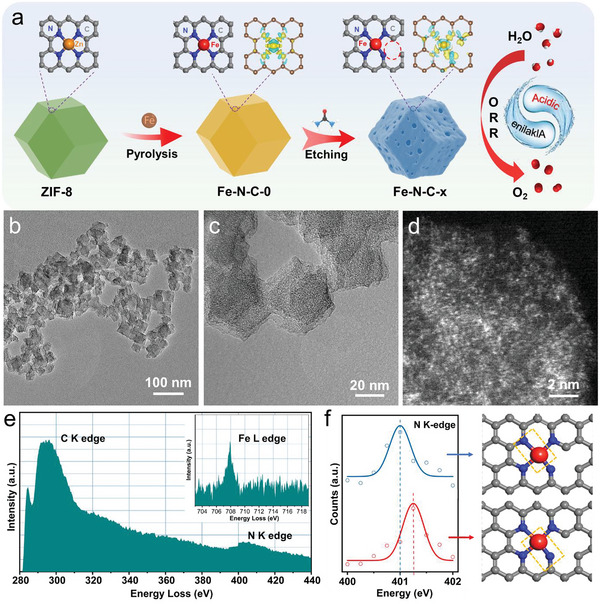
Synthetic procedures and structural analysis of Fe–N–C‐2. a) Schematic of the synthesis strategy of Fe–N–C‐*x*. b) TEM image. c) HRTEM image. d) HAADF‐STEM image. e) EELS analysis of selected paired spots of Fe–N–C–2 in HAADF‐STEM images. f) Schematic diagram of the standard peak analysis of Fe–N–C‐2 in N EELS and its corresponding FeN bond.

The preparation of Fe–N–C catalysts with broken electronic symmetry of Fe─N_4_ moieties was carried out on a Fe‐modified ZIF‐8 precursor via pyrolysis combining urea etching. In a typical procedure, as shown in **Figure** **1**a, Fe‐replaced ZIF precursor, Fe‐Urea@ZIF‐8‐*x* (*x* represents the molar ratio of iron to urea) was first synthesized from 2‐methylimidazole, Fe(C_5_H_5_)_2_, Zn (NO_3_)_2_, urea, and methanol solution at the room temperature. For comparison, ZIF‐8 prepared without the addition of Fe(C_5_H_5_)_2_ and urea and Fe‐ZIF‐8 without the addition of urea were also synthesized under the same conditions. Then, ZIF‐8, Fe‐ZIF‐8, and Fe‐Urea@ZIF‐8‐*x* were carbonized at 900 °C in an inert atmosphere to prepare the corresponding N─C (perfect N‐doped carbon), Fe–N–C‐0 (Fe–N–C without etching), and Fe–N–C‐*x* catalysts. The details of the synthesis of various Fe–N–C catalysts can be found in the Supplementary Information. The accurate contents of each element were examined by an inductively coupled plasma emission spectrometer (ICP‐OES) (Table S1, Supporting Information). The Fe content in the Fe–N–C‐2 was 4.5 wt.%. Owing to the etching of carbon by urea, the Fe content increased with the increase of urea addition amount.

The structural evolutions of the Fe–N–C‐*x* catalysts showed a good correlation with *x*, the Fe to urea ratio. The x‐ray powder diffraction (XRD) patterns demonstrate that the Fe‐doped ZIF (Fe‐ZIF‐8) has the same crystal structure with the perfect ZIF‐8 (Figure S1, Supporting Information). Without the addition of urea, the obtained Fe–N–C‐0 catalyst after the pyrolysis of Fe‐ZIF‐8 displayed a complete dodecahedron shape with a smooth surface and a firm interior as that of the N─C sample (Figure S3, Supporting Information). The addition of urea clearly changed the morphology and the microstructure of the Fe–N–C‐*x* materials. It is interesting that Fe–N–C‐2 generated sufficient carbon defects but maintained the integrity of the overall structure (Figure 1b,c; Figure S2, Supporting Information). Higher urea etching agent in Fe–N–C‐3 and Fe–N–C‐4 resulted in the collapse of the dodecahedron morphology, due to the formation of excess amount of carbon vacancies ruined the carbon frameworks (Figures S5 and S6, Supporting Information). Even a ribbon‐like structure with obvious Fe agglomeration in the form of nanoparticles was observed for Fe–N–C‐5 (Figure S7, Supporting Information). However, less urea amount in Fe–N–C‐1 cannot form enough carbon vacancies within the framework (Figure S4, Supporting Information).

High‐angle annular dark‐field scanning transmission electron microscope (HAADF‐STEM) was employed to accurately determine the spatial distribution of the Fe atoms in the Fe–N–C‐2 catalyst (Figure 1d). It can be clearly identified that high‐density single Fe atoms (brighter spots) were uniformly dispersed in the porous carbon substrate. Meanwhile, the electron energy loss spectroscopy (EELS) analysis for Fe and N were carried out on some selected spots on Fe–N–C‐2 by placing the electron probe onto isolated bright spots (Figure 1e). The EELS spectrum not only proved the existence of stable Fe─N_4_ configuration throughout the catalyst but also provided electronic states of the elements. The EELS analysis was also performed on Fe–N–C‐0. As shown in Figure S8 (Supporting Information), only one characteristic peak of N at 401 eV was identified for the Fe–N–C‐0 catalyst, which represents the standard Fe─N bonds in the Fe─N_4_ configuration. For the Fe–N–C‐2 catalyst, two characteristic peaks of N were identified (Figure 1f), in which the N peak located at 401 eV represents the standard Fe─N bond for Fe─N_4_ configurations, while the shifting of the characteristic peak to 401. 26 eV indicates the increased bond energy of Fe─N bond, which is attributed to the broken of the nearest C─N bonds adjacent to the Fe─N_4_ with the formation of carbon vacancies.^[^ ^36^, ^37^ ^]^ This result directly demonstrates the selective breaking of the C─N bonds adjacent to Fe─N_4_, which leads to the generation of asymmetric distribution of the electronic density of the Fe─N_4_ moieties.

The elemental mapping image was recorded by using energy dispersive X‐ray spectroscopy analysis (EDS), as displayed in Figure S9 (Supporting Information), suggesting that the N and Fe elements were evenly dispersed into the carbon matrix. Therefore, Fe–N–C‐0 has a completely symmetrical Fe─N_4_ structure without the disturbance in the electronic structure. Fe–N–C‐2 etched with an appropriate amount of urea generates suitable carbon vacancies to endow the Fe─N_4_ structures with local asymmetry but without the collapse of the overall framework. The excessive urea etching (Fe–N–C‐5) results in not only the collapse of the carbon frameworks, but also the agglomeration of the Fe‐atoms into Fe nanoparticles.

### 1.2 Identification of Atomic Structure

**Figure 2:**
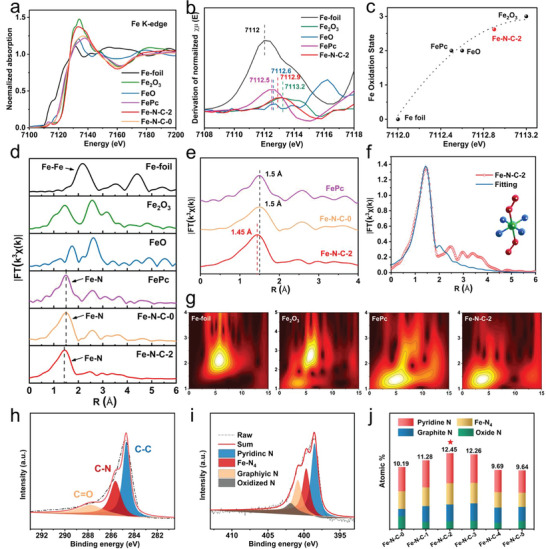
Chemical state and atomic local structure of Fe–N–C‐2. a) Fe K‐edge XANES spectra of Fe–N–C‐0, Fe–N–C‐2 and reference samples Fe foil, FePc, FeO, and Fe_2_O_3_. b) First‐derivative XANES curves of Fe–N–C‐2 and the references. c) Correlation between the Fe oxidation state and the energy position of the XANES spectrum, determined as the first maximum of the first derivative spectrum of Fe–N–C‐2 and different iron reference compounds. d) Fourier transform plots of the EXAFS spectra of Fe–N–C‐0, Fe–N–C‐2 Fe foil, FePc, FeO, and Fe_2_O_3_. e) The enlarged Fourier transform plots of the EXAFS spectra of Fe–N–C‐0, Fe–N–C‐2, and FePc in (d). f) Fe K‐edge Fourier transform EXAFS spectrum and its fitting, with the O_2_‐Fe‐N_4_ model presented in the inset, wherein the green, blue and red balls represent Fe, N, and O atoms, respectively. g) Wavelet transform of the K^3^‐weighted EXAFS data of Fe foil, Fe_2_O_3_, FePc and Fe–N–C‐2, respectively. h) XPS spectra of C *1s* in Fe–N–C‐2. i) XPS spectra of N *1s* in Fe–N–C‐2. j) The contents of graphitic N, pyrrolic N, Fe‐N_4_ and pyridinic N for Fe–N–C‐*x*.

It is clear that the Fe–N–C‐2 catalyst has the most suitable carbon vacancies to generate electronic asymmetry in the Fe─N_4_ moieties but does not damage the carbon framework to support the atomically dispersed Fe atoms. The atomic‐level local electronic structures of the Fe–N–C‐2 catalyst were further investigated by X‐ray absorption near‐edge structure (XANES) and extended X‐ray absorption fine structure (EXAFS) analyses. The XANES and FT‐EXAFS spectra of the Fe–N–C‐0 catalyst were provided for comprising with those of Fe–N–C‐2 to show the differences of the atomic configurations. As shown in **Figure** **2**a, the position of the Fe K‐edge of Fe–N–C‐0 is basically the same as that of FePc, indicating that the valence state of Fe in Fe–N–C‐0 is +2. The Fe K‐edge position of the Fe–N–C‐2 catalyst is located between those of FePc and Fe_2_O_3_, indicating a Fe valence state between +2 and +3. Comparison of the first derivative XANES of Fe–N–C‐2 with the references also indicates that the Fe atoms in the catalyst should be at a valence state between +2 and +3. By fitting the oxidation state of the Fe element in Fe–N–C‐2 with the references, the Fe valence state in Fe–N–C‐2 is ≈2.6 (Figure 2b and Figure 2c), which indicates that the oxidation state of Fe is between divalent and trivalent, namely, Fe^2+^ and Fe^3+^ coexist in the Fe–N–C‐2 catalyst. It is usually that only Fe^3+^─N_4_ configuration can be obtained in the Fe–N–C catalysts.^[^ ^29^, ^31^ ^]^ One reason is that Fe^2+^ in the iron source can be easily oxidized into Fe^3+^ during the synthesis of the materials, which leads to the formation of Fe^3+^─N_4_ configuration. It has been reported that, however, the Fe^2+^─N_4_ configuration is much more active toward ORR catalysis than the Fe^3+^─N_4_ configuration.^[^ ^38^ ^]^ In this work, during the preparation of Fe–N–C‐2, the urea etching causes selective break of the C─N bond adjacent to the Fe─N_4_ sites and the asymmetric charge distribution surrounding Fe─N_4_ leads to the increased electronic density and thus the co‐existence of Fe^2+^─N_4_ configurations (Fe^2+^─N_4_Vc). The electronic asymmetry caused by carbon vacancy (Fe^2+^─N_4_Vc) was further confirmed by DFT calculations. As shown in Figure S10 (Supporting Information), with the breaking of C─N bond adjacent to the Fe^3+^─N_4_ sites, the charge number of Fe decreases from 1.31 for the entire Fe─N_4_─C configuration to 1.25 for the Fe─N_4_Vc configuration with a nearest carbon vacancy and obvious charge asymmetric distribution is observed in the Fe^2+^─N_4_Vc configuration. Therefore, the introduction of the nearest carbon vacancies causes the transformation of Fe^3+^ into Fe^2+^ and the asymmetric distribution of the electronic densities of the Fe─N_4_ moieties.

Further information about the structural configurations can be read from the pre‐edge spectra and the Fourier transformed EXAFS spectra. As shown in Figure S11 (Supporting Information), the pre‐edge peak for Fe–N–C‐2 at ≈7115 eV was dissimilar from those of Fe foil, FePc, FeO, and Fe_2_O_3_ in terms of strength, form, and location, and can be ascribed to Fe 1*s*→3*d* transition and quadruple allowed transition. Therefore, it has been thought of as a potential indicator of the Fe─N_4_ rectangular planar structure with a deviation from the rectangular plane of Fe─N_4_ (≈ 7118 eV), indicating the presence of axial ligands. In Figure 2d, the Fourier transformed EXAFS profiles for Fe–N–C‐0 and Fe–N–C‐2 showed a main peak at 1.5 Å in R space, which is very close to the distance of the FePc reference sample and can be assigned to the Fe─N(O) configuration. Unlike the Fe foil, the Fe–Fe peak at ≈2.2 Å was not observed in the Fe–N–C‐2 sample, which provides enough proof for atomically scattered Fe in the obtained Fe–N–C‐2. Simultaneously, we found that the positions of Fe─N peaks in FePc and Fe–N–C‐0 were at 1.5 Å, indicating that the Fe─N bond in Fe–N–C‐0 is the same as that of FePc, with a standard Fe─N bond length. However, the Fe─N peak shifted obviously to a low‐R position from 1.5 to 1.45 Å in Fe–N–C‐2, implying that the Fe─N bond length is shrank and the local structure of the Fe─N_4_ active site is changed (Figure 2e).^[^ ^39^ ^]^ For Fe with a coordination number of 6, such as FeO, it has a mean bond length of ca. 2.0 Å. In the Fe–N–C structure, N atoms are four‐fold coordinated with the single Fe atom. We fitted the Fe K‐edge Fourier transform EXAFS profile of Fe–N–C‐2 by using a structure of Fe─N_4_ adsorbed two O_2_ molecules. As shown in Figure 2f, the atomic configuration of Fe atom can be described as a Fe─N_4_ plane with two oxygen molecules at the axial direction. The EXAFS wavelet transforms (WT) analysis of Fe–N–C‐2 and the references (Fe foil, FePc, FeO, and Fe_2_O_3_) were illustrated in Figure 2g, which provides not only redial distance resolution but also k‐space resolution. The WT analysis of the Fe–N–C‐2 structure exhibits only one intensity maximized at 4 Å, indicating that only Fe─N_4_‐C_x_/O exists and metallic crystallites are not formed within the Fe–N–C structure like those in Fe_2_O_3_.^[^ ^40^ ^]^ This is also unambiguous evidence of the atomically dispersed Fe‐atoms within the Fe–N–C structure. Combining the EELS and XANES/EXAFS analyses, we are confident to conclude that atomically dispersed Fe atoms at a Fe^2.6+^─N_4__‐_Vc structure have been successfully obtained with an asymmetric distribution of the electronic density surrounding the Fe active centers.

Besides the atomic level characterizations on the structural configurations, the overall phase formation and surface chemistry states were examined. The XRD patterns of all Fe–N–C‐*x* samples (Figure S12a, Supporting Information) demonstrated the existence of graphitized carbon but without of the detection of any Fe‐containing phases, indicating the atomically dispersed Fe atoms in the samples. By etching with urea, Fe–N–C‐2 exhibited higher Brunauer–Emmett–Teller (BET) surface area (963.0587 m^2^ g^−1^) than that of the Fe–N–C‐0 sample (879.5574 m^2^ g^−1^) (Figure S12b, Supporting Information) and a wider pore size distribution (Figure S12c, Supporting Information), due to the introduction of abundant micropores. The relative intensity ratio (*I*_D_/*I*_G_) of the D band and the G band in the Raman spectra can provide evidence for the graphitization degree and the defect content of carbon. The results in Figure S13 (Supporting Information) showed that the *I*_D_/*I*_G_ values of Fe–N–C‐*x* gradually increased with the content of etching reagent, indicating the increased etching effect and more defects in Fe–N–C‐*x*. In our previous study,^[^ ^41^ ^]^ we have demonstrated that only a proper defect concentration can lead to the optimal adsorption of intermediates and thus the best catalytic performance, so that the Fe–N–C‐2 sample with appropriate defect concentration could exhibit the best ORR performance. For the purpose of examining the surface chemical states of the produced catalysts, X‐ray photoelectron spectroscopy (XPS) was employed (Figure S14, Supporting Information) and the obtained element contents were listed in Table S2 (Supporting Information). In the high‐resolution C *1s* spectra, C─C, C─N, and C═O species were identified (Figure 2h; Figure S15a–e, Supporting Information). It shows that the content of the C─N bonds decreased with the etching degree or the urea amount, indicating the selective cleavage of C─N bonds by urea (Figure S15f, Supporting Information). The N content, however, first increased but then decreased with the increase of urea amounts. There are four types of N existing in the Fe–N–C structure: pyridine N (398.5 eV), Fe─N_4_ (399.6 eV), graphite N (400.9 eV), and oxidized N (402.9 eV) (Figure 2i; Figure S16, Supporting Information). The contents of N associating with the active Fe‐N_4_ moieties reached the highest value for the Fe–N–C‐2 catalyst, further confirmed that the over‐etching by urea ruined not only the overall carbon framework but also the active site numbers (Figure 2j).

### 1.3 Electrochemical Oxygen Reduction Performance

**Figure 3:**
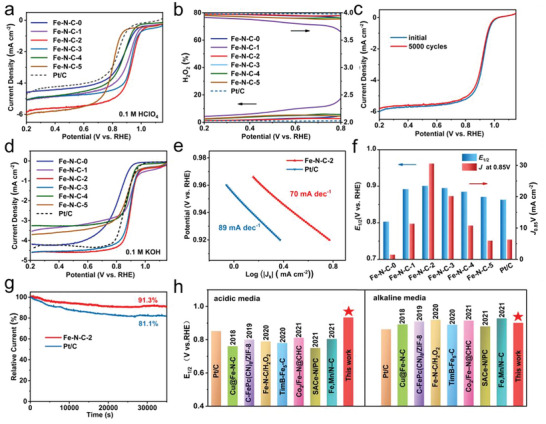
ORR activity of Fe–N–C‐2. a) LSV curves of Fe–N–C‐*x* and Pt/C in O_2_‐staurated 0.1 m HClO_4_ solution at 1600 rpm. b) H_2_O_2_ yield and electron transfer number of Fe–N–C‐*x* and Pt/C versus potential. c) Stability test of Fe–N–C‐2 and Pt/C in 0.1 m HClO_4_. d) LSV curves of Fe–N–C‐*x* and Pt/C in O_2_‐staurated 0.1 m KOH solution at 1600 rpm. e) Tafel plots of Fe–N–C‐2 and Pt/C in 0.1 m KOH. f) *J*_k_) at 0.85 V and *E*_1/2_ for these catalysts. g) Chronoamperometric responses of Fe–N–C‐2 and Pt/C at 900 rpm for 35 000 s in 0.1 m KOH. h) Comparison of *E*_1/2_ of various full pH catalysts.^[^ ^42^, ^43^, ^44^, ^45^, ^46^, ^47^, ^48^ ^]^

To validate our idea on improving the catalytic activity of Fe─N_4_ moieties by electronic asymmetry engineering, the electrochemical performance of Fe–N–C‐*x* toward ORR was examined. The electrocatalytic ORR activity of the catalysts were evaluated by using a rotating disk electrode (RDE) technology in a 0.1 m HClO_4_ electrolyte solution saturated with O_2_. For comparison, a commercial Pt/C catalyst (20% Pt, Fuelcellstore) was also used as a referential catalytic electrode. The linear sweep voltammetry (LSV) results showed that the Fe‐free N─C catalyst presented very poor catalytic activity toward ORR, while the defect‐free Fe–N–C‐0 catalyst displayed a high *E*_1/2_ value of 0.862 V (Figure S17, Supporting Information), indicating that the existence of Fe─N_4_ moieties is essential for active ORR catalysis. **Figure** **3**a displays the effect of carbon‐defects on the ORR catalysis performance. Table S3 (Supporting Information) lists the onset potential (*E*_onset_), the *E*_1/2_, and the steady‐state limiting current density of the obtained catalysts and the benchmark Pt/C (20 wt.%). It is obvious that the Fe–N–C‐2 catalyst with proper carbon‐defect and introduced electronic asymmetry exhibited the highest ORR activity, which reached a high *E*_onset_ value of 1.015 V and a high *E*_1/2_ value of 0.934 V, both of which are superior to those of the commercial Pt/C referential catalyst (*E*_onset_ = 1.002 V, *E*_1/2_ = 0.862 V) and the previously reported non‐noble metal catalysts. The LSV curves for the Fe–N–C‐2 catalyst were also recorded at different speeds (Figure S18a, Supporting Information) with the calculated Koutecky–Levich (K–L) curves (Figure S18b, Supporting Information). The result shows that the electron transfer number (n) of Fe–N–C‐2 was >3.91 in the range of 0.3 to 0.7 V, indicating that a 4e^−^ reaction process and a highly selective oxygen reduction to hydroxide reaction were occurred on the surface of Fe–N–C‐2 catalyst. As shown in Figure 3b, the H_2_O_2_ yield of Fe–N–C‐2 was very low over 0.2–0.8 V as the ORR behavior examined by the rotating ring disk electrode (RRDE). It is very impressive that the Fe–N–C‐2 catalyst presented excellent catalytic durability in the acidic solution, and the *E*_1/2_ value of Fe–N–C‐2 had almost no negative shift after 5000 cycles (Figure 3c). The Tafel slope for Fe–N–C‐2 was 70 mV·dec^−1^, which is smaller than that of the Pt/C catalyst (89 mV·dec^−1^), indicating that the electron transfer speed is much faster in Fe–N–C‐2 (Figure S19, Supporting Information). Meanwhile, the current density in the kinetic range (*J*_k_ at 0.85 V) also shows that the Fe–N–C‐2 catalyst exhibited the highest activity compared with Pt/C and other Fe–N–C catalysts (Figure S20, Supporting Information). The Fe–N–C‐2 catalyst also presented excellent durability in the 0.1 m HClO_4_ electrolyte. The current density retention of 87.3% was recorded after continuous reaction of 35 000 s for Fe–N–C‐2, while it was only 63.8% for the Pt/C catalyst (Figure S21, Supporting Information), demonstrating the excellent performance of the Fe–N–C‐2 catalyst under an acidic condition.

The catalytic performance of the Fe–N–C catalysts under alkaline conditions was also examined in the 0.1 m KOH electrolyte. The CV curves of the Fe–N–C‐*x* catalysts showed a high double‐layer capacity current close to a rectangle in a N_2_ saturated solution, and a sharp cathode peak in the O_2_ saturated solution, indicating that they have potentially high activity. In the CV scans, the Fe–N–C‐2 showed the highest reduction peak at 0.90 V in the O_2_‐saturated KOH solution, which demonstrates the excellent ORR activity of the catalyst under the alkaline condition (Figure S22, Supporting Information). From the LSV results (Figure 3d), a similar activity trend for the catalysts in the KOH solution was reflected as those in the acidic solution. Table S4 (Supporting Information) lists the *E*_onset_ values, the *E*_1/2_ value, and the steady‐state limiting current density of the obtained catalysts and the benchmark Pt/C (20 wt.%). The Fe–N–C‐2 catalyst exhibited the highest activity with a super high *E*_onset_ value of 1.015 V and an *E*_1/2_ value of 0.901 V, while the Pt/C catalyst showed only *E*_onset_ = 1.01 V and *E*_1/2_ = 0.853 V. The electron transfer number during the catalysis reaction for Fe–N–C‐2 was >3.95 in the range of 0.3 to 0.7 V as estimated from the LSV measurements performed at different speeds (Figure S23, Supporting Information) and the low H_2_O_2_ yield (Figure S24, Supporting Information), indicating that the Fe–N–C‐2 catalyst with asymmetric electronic distribution also facilitates the 4e^−^ reaction process in an alkaline condition. The Tafel slope of Fe–N–C‐2 was 78 mV·dec^−1^, which is slightly higher than that in an acidic solution but still smaller than that of Pt/C catalyst (87 mV·dec^−1^) (Figure 3e). The current density (Jk) in the kinetic range (*J*_k_ = 0.85 V) confirms that the Fe–N–C‐2 catalyst had the highest activity among the examined catalysts (Figure 3f). It means that the ORR catalytic reaction on the Fe–N–C catalysts is a bit sluggish in the alkaline condition, but yet much higher than that on the Pt/C catalyst.

The stability of Fe–N–C‐0 and Fe–N–C‐2 catalysts were examined up to 5000 CV cycles under the same test condition. As shown in Figures S25 and S26 (Supporting Information), after the stability tests, the LSV curve of Fe–N–C‐0 showed a negative shift of 5 mv, while almost no negative shift was observed in Fe–N–C‐2, because the anchoring effect of carbon defects to the Fe single atoms in Fe–N–C‐2 increases the stability of Fe─N_4_ active sites and then improves the ORR stability of materials. After continuous reaction for 35 000 s, compared with a decay of 18.9% for the Pt/C catalyst, the Fe–N–C‐2 catalyst exhibited a higher original current density retention rate of 91.3%, further confirming its good durability (Figure 3g). The methanol tolerance test was further carried out. It is found that there was almost no change detected in the current with the addition of methanol, but an obvious current jump was observed for Pt/C, indicating that the Fe–N–C‐2 catalyst has a good methanol tolerance (Figure S27, Supporting Information). At the same time, Fe–N–C‐2 showed good performance in both acidic and alkaline solutions. The surface elemental compositions before and after the stability tests were determined from the XPS analyses, as shown in Table S5 (Supporting Information). Slight reduction of Fe and N elements were identified, which may be due to the partial consumption of the Fe─N_4_ active sites involved in reaction during the stability test. For N element in Fe–N–C‐2 tested after 5000 cycles, as shown in Figure S28 (Supporting Information), the peak intensity of Fe─N_4_ decreased slightly. The slight drifting of the chemical states of Fe–N–C‐2 is ascribed by the slightly consumed Fe─N_4_ active sites and the 8.7% performance decay. However, comparing with the reference catalysts, the Fe–N–C‐2 catalyst still has good stability as a whole.

The electrochemical active surface area (ECSA) gives the information of specific catalytic activity of the catalyst and is a key parameter in evaluating the performance of electrodes, which can be obtained by measuring the corresponding double‐layer capacitances (*C*_dl_) derived from the linear correlation between scan speeds and current density at a non‐Faradaic potential. As shown in Figures S29–S31 (Supporting Information), the notably larger ECSA for Fe–N–C‐2 tallies with its greater ORR activities in a universal pH range.

Based on the above measured results, it is clear that the ORR catalytic activites of Fe–N–C‐2 under both acidic and alkaline conditions outperform other Fe–N–C catalysts and the commercial Pt/C catalyst. To well position the electronic asymmetry engineered Fe–N–C‐2 catalyst in current ORR catalysts, we compared performance with the ORR catalysts employed in the full pH range reported in recent years (Figure 3h; Table S6, Supporting Information). Under acidic conditions, the Fe–N–C‐2 catalyst is significantly superior to other reported catalysts, showing the highest half‐wave potential. Under alkaline conditions, Fe–N–C‐2 catalyst is also comparable to the top level of the catalysts reported in recent years. Consequently, it can be concluded that the electronic asymmetry engineered Fe–N–C catalyst possesses extremely high ORR catalytic activity in a universal pH range and has promising potential in practical ORR catalysis applications.

### 1.4 Catalytic Mechanisms Investigation

**Figure 4:**
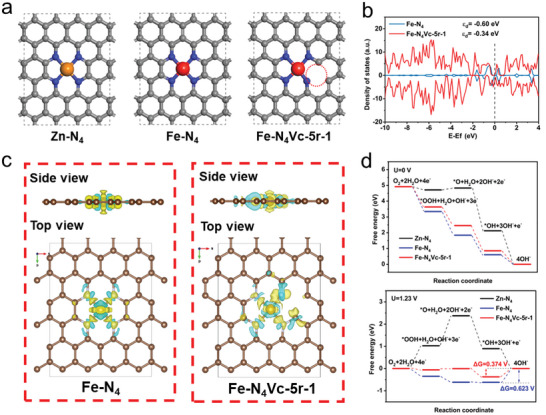
Theoretical ORR activity of Fe–N–C‐2. a) Schematic diagram of the atomic configuration of Zn‐N_4_, Fe─N_4_, and Fe─N_4_Vc‐5r‐1 corresponding to N─C, Fe–N–C‐0, Fe–N–C‐2. b) The PDOS of Fe–d orbitals in Fe–N_4_ (above) and Fe─N_4_Vc‐5r‐1 (below). c) The computed differential charge density between Fe─N_4_ and Fe─N_4_Vc‐5r‐1 from top and side views. Yellow and blue bubbles separately represent the electron and hole charges. d) ORR free energy diagrams in Zn─N_4_ (black line), Fe─N_4_ (blue line), and Fe─N_4_Vc‐5r‐1 (red line) at *U* = 0 and *U* = 1.23 V.

To study the mechanism of Fe–N–C‐2 catalyst for boosting the ORR activity, density functional theory (DFT) calculations were performed on the carbon vacancy induced electronic distribution asymmetry of the Fe─N_4_ moieties and its effect on the catalytic behaviors. The local atomic configurations of the active centers of Zn─N_4_, Fe─N_4_, and Fe─N_4_Vc (Fe─N_4_Vc‐5r‐1) corresponding to N─C, Fe–N–C‐0, and Fe–N–C‐2 were constructed and optimized (**Figure** **4**a). It is apparent that, in the Fe─N_4_Vc structure, the Fe─N bond length close to carbon vacancy is shorter than the regular Fe─N bond in Fe─N_4_ moieties, attributed to the broken of the adjacent C─N bonds with the introduction of an adjacent carbon vacancy. We also studied the Fe─N_4_ structures with the carbon vacancies to explore the effect of position and the numbers of carbon vacancies on the electronic structure of the Fe─N_4_ moieties (Figure S32, Supporting Information). Figure 4b presents the projected density of states (PDOS) of Fe atoms in Fe‐N_4_ structure and the Fe─N_4_Vc structure. It can be found that the spin‐up and the spin‐down DOS are symmetric for Fe─N_4_ but become asymmetric for the Fe─N_4_Vc. The introduction of carbon vacancy also leads to change of d‐band center (*e*_d_). The *e*_d_ value for Fe in Fe‐N_4_ is −0.60 eV, while it moves to a higher energy level (−0.34 eV) in Fe─N_4_Vc. According to the d‐band center theory, the higher d‐band center value of Fe─N_4_Vc indicates the less the anti‐bonding electron filling below the Fermi level, which leads to higher activity in the ORR process.^[^ ^49^, ^50^ ^]^

The differential charge density maps of Fe─N_4_ and Fe─N_4_Vc‐5r‐1, where the yellow and blue bubbles represent the electron and hole charges, respectively (Figure 4c), clearly demonstrate that the formation of one adjacent carbon vacancy with the break of the C─N bond leads to the redistribution of electronic density on the defect surface, and more positive charges are accumulated near the Fe site from the carbon beside the vacancy, which result in an asymmetrical charge density distribution and a partially reduced Fe state, and then the significantly reduced energy barrier of the ORR process. It is interesting that this electronic asymmetry is not interfered by the adsorption of O_2_ (Figure S33, Supporting Information), which is the essential step to catalyze the subsequent O═O bond breaking. The calculated binding energy of Fe─N_4_Vc‐5r‐1 to O_2_ is only −1.66 623 eV, much lower than that of the O_2_ adsorption on the Fe─N_4_ site (−2.27 185 eV).

It shows that high electron density of the Fe─N_4_ structure causes an excessively strong binding of the oxygen intermediates, which is caused by an upshifting of the *d*_z2_ metal orbital and an increase in the molecular hardness, or the distance between *d*_z2_ and the molecular oxygen energy level, but the asymmetric electronic density of the Fe─N_4_Vc structure optimizes the adsorption toward O_2_ and the intermediates and thus lowers the energy barrier for ORR.^[^ ^51^ ^]^ Figure 4d and Figure S34 (Supporting Information) display the Gibbs free energy profile of ORR catalysis steps on different structures at *U* = 0 V. Both the normal Fe─N_4_ moieties and Fe─N_4_Vc structures showed a consistent downhill energy pathway, indicating a thermodynamically favorable process on their surfaces. At a potential of 1.23 V, however, the rate determination steps (RDS) of the Fe─N_4_ and Fe─N_4_Vc structures presented an uphill free energy profile ascribed by the fourth electron transfer step (*OH + e^−^ → OH^−^). Among Fe─N_4_Vc with different configurations,Fe–N_4_‐Vc‐5r‐1 presents the smallest uphill free energy (0.374 V) and thus the highest activity, which can greatly promote the ORR reaction. The DFT calculations thus confirm that the electronic asymmetry of Fe─N_4_Vc not only provides a proper d‐band center for favorable ORR catalysis but also optimizes the O_2_ adsorption behavior during the catalysis, and thus significantly enhances the ORR catalytic performance of the Fe–N–C catalysts.

### 1.5 Zinc–Air Battery Performance

**Figure 5:**
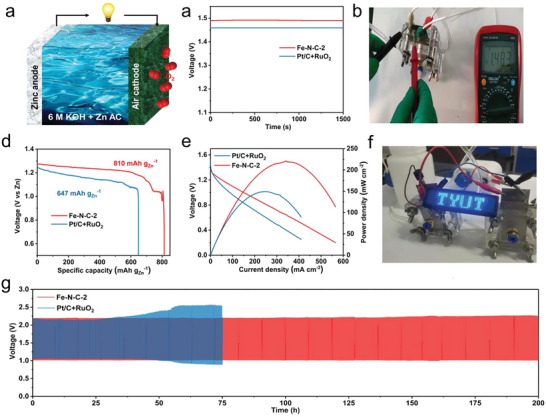
ZAB performance. a) Schematic configuration of the homemade ZAB. b) OCV of the ZAB based on Fe–N–C‐2 and Pt/C+RuO_2_. c) Voltage measured by a multimeter. d) Specific discharging capacities of the cells with Fe–N–C‐2 and Pt/C+RuO_2_ cathodes at 10 mA cm^−2^, respectively. e) The corresponding power density curves of Fe–N–C‐2 and Pt/C+RuO_2_. f) ZAB successfully lights up the LED screen. g) Long‐term cycling performance of ZABs based on Fe–N–C‐2 and Pt/C+RuO_2_ at a current density of 10 mA cm^−2^.

The effectiveness of Fe–N–C‐2 catalyst in renewable energy devices is evaluated by incorporating it into the cathode of a ZAB (**Figure** **5**a). As a comparison, commercial Pt/C+RuO_2_ with a mass ratio of 1:1 was assembled as the air cathode. As shown in Figure 5b, the Fe–N–C‐2‐based ZAB exhibited an open‐circuit voltage (OCV) of 1.483 V, superior to 1.458 V reached in the Pt/C+RuO_2_‐based ZAB. The real‐time voltage measured with a multimeter was 1.483 V (Figure 5c). At a fixed current density of 10 mA cm^−2^, no significant change of the discharge voltage was observed after 166 h (Figure S35, Supporting Information). The specific capacity of Fe–N–C‐2‐based ZAB was 810 mAh g^−1^, which is also superior to 647 mAh g^−1^ achieved in the Pt/C+RuO_2_‐based ZAB (Figure 5d). Moreover, the maximum power density was 218 mW cm^−2^ for the ZAB based on the Fe–N–C‐2 catalyst, outperforming149 mW cm^−2^ for the Pt/C+RuO_2_‐based ZAB (Figure 5e). As shown in Figure 5f, a serial connection of two Fe–N–C‐2‐based ZABs successfully lit up an LED display, indicating the practical application potential of the Fe–N–C‐2 catalyst in real ZABs. The cycle performance of ZAB based on both Fe–N–C‐2 and Pt/C+RuO_2_ catalysts were examined through constant current charge and discharge cycles with an interval of 20 min at a fixed current density of 10 mA cm^−2^. For the device based on Pt/C+RuO_2_ catalyst, the discharge voltage started to drop after 25 h and lost its capacity within 75 h. For the ZAB based on Fe–N–C‐2 catalyst, the voltage attenuation during the charging and discharging was negligible even after 200 h (600 cycles) (Figure 5g). Therefore, the Fe–N–C‐2 catalyst with an asymmetric electronic distribution on the Fe‐N_4_ sites induced by an adjacent carbon vacancy not only provides high‐efficient ORR catalytic activity with outstanding environmental durability in a universal pH range, but also demonstrates practical application potential in real‐world ZZAB devices with outperforming capacity, cycling performance, and long‐term stability.

## 2 Conclusion

In summary, an atomically dispersed Fe–N–C‐Vc catalyst with an asymmetric electronic distribution on the Fe─N_4_ moieties induced by an adjacent carbon vacancy was fabricated from ZIF‐8 framework assisted with urea‐etching. The adjacent carbon vacancy results in significantly unbalanced electronic density distribution of the Fe─N_4_ moieties, which leads to an optimizing d‐band center for favorable O_2_ and intermediates adsorption and lowers the energy barrier of the catalytic reactions. Benefiting from the well dispersed Fe^2+^─N_4_ active sites with asymmetric charge distribution states, the Fe–N–C‐Vc catalyst delivered a superior catalytic activity toward ORR in both acidic and alkaline conditions compared to the commercial Pt/C catalyst and the recently reported Fe‐based ORR catalysts. When applied as the cathode catalyst for rechargeable flexible ZABs, the Fe–N–C catalyst exhibited an OCV of 1.483 V, a specific capacity of 810 mAh g^−1^ at 10 mA cm^−2^, a peak power density of 218 mW cm^−2^, and long‐term stable cycling for 200 h, which significantly outperform the ZAB examined under the same conditions with the noble metal‐based Pt/C+RuO_2_ catalyst. This work provides a high‐efficient non‐noble metal‐based catalysts with high activity and durability for ORR catalysis in the full pH range and practical application potential and offers insights into designing Fe–N–C‐based catalysts via proper electronic asymmetry engineering of the moieties.

## 3 Experimental Section

### Preparation of Fe‐ZIF‐8

Fe‐ZIF‐8 was synthesized according to the previously reported method with some modifications. Here, 2.628 g 2‐methylimidazole, 1.188 g Zn (NO_3_)_2_·6H_2_O and 0.35 g Fe(C_5_H_5_)_2_ were added to 50 mL methanol solution and stirred at room temperature until completely dissolved, then the stir was continued for 24 h. The precipitate was collected by centrifugation, and then washed with methanol several times until the supernatant became clear. Finally, the prepared product was vacuum treated at 60 °C overnight before used. Fe‐ZIF‐8 with a particle size of 40 nm was obtained.

### Preparation of Fe–N–C‐*x* Catalysts

2.628 g 2‐methylimidazole, 1.188 g Zn (NO_3_)_2_·6H_2_O and 0.35 g Fe(C_5_H_5_)_2_ were added to 50 mL methanol solution and stirred at room temperature until completely dissolved, and then the stir was continued for 24 h. The precipitate was collected by centrifugation, and then washed with methanol several times until the supernatant became clear. Finally, the prepared product was vacuum treated at 60 °C overnight before used. In Ar atmosphere, the temperature was increased to 900 °C at 5 °C min^−1^ and the temperature was maintained for 3 h to obtain Fe–N–C‐0 sample. The preparation of Fe–N–C‐*x* was the same as above, adding different content of urea during the synthesis process, the molar ratio of Fe(C_5_H_5_)_2_ to urea (1:0.15, 1:0.8, 1:1.5, 1:4, 1:8) corresponded to Fe–N–C‐1, Fe–N–C‐2, Fe–N–C‐3, Fe–N–C‐4, and Fe–N–C‐5.

## Conflict of Interest

The authors declare no conflict of interest.

## Supporting information

Supporting Information

## Data Availability

The data that support the findings of this study are available in the supplementary material of this article.
