# Supplementary material for: Electronic Asymmetry Engineering of Fe–N–C Electrocatalyst via Adjacent Carbon Vacancy for Boosting Oxygen Reduction Reaction
Source: Adv Sci (Weinh). 2023 Sep 26;10(32):2305194. doi: 10.1002/advs.202305194 (PMC10646226; doi:10.1002/advs.202305194)
Supplement: Supplementary file 1 — Supporting Information [file ADVS-10-2305194-s001.pdf]

ADVANCED SCIENCE
Open Access

Supporting Information

for *Adv. Sci.*, DOI 10.1002/advs.202305194

Electronic Asymmetry Engineering of Fe–N–C Electrocatalyst via Adjacent Carbon Vacancy for Boosting Oxygen Reduction Reaction

Huanlu Tu, Haixia Zhang, Yanhui Song, Peizhi Liu, Ying Hou, Bingshe Xu, Ting Liao, Junjie Guo* and Ziqi Sun**

Supporting Information

Electronic Asymmetry engineering of Fe-N-C electrocatalyst with boosting oxygen reduction reaction activity

*Huanlu Tu, Haixia Zhang, * Yanhui Song, Peizhi Liu, Ying Hou, Bingshe Xu, Ting Liao, Junjie Guo, * and Ziqi Sun**

Experimental Section

Materials and reagent. All chemicals are from commercial sources and used without further treatment. The specifics are as follows: zinc nitrate hexahydrate ($Zn(NO_3)_2·6H_2O$, 99%, Shanghai Aladdin Biochemical Technology Co., Ltd.), 2-methylimidazole ($C_4H_6N_2$), 98%, Shanghai Aladdin Biochemical Technology Co., Ltd.), ferrocene ($Fe(C_5H_5)_2$, 98%, Shanghai Aladdin Biochemical Technology Co., Ltd.), urea (CH_4N_2O, Shanghai Aladdin Biochemical Technology Co., Ltd.) , Anhydrous methanol (CH_3OH, ≥99.5%, Sinopharm Chemical Reagent Co., Ltd.), anhydrous ethanol (C_2H_5OH, ≥99.7%, Tianjin Damao Chemical Reagent Factory), Nafion (5 wt%, Shanghai Chuxi Industrial Co., Ltd.) and Pt/C (20 wt%, Alfa Aesar).

Characterization of the samples. A scanning electron microscope (SEM) was used to study the morphology of each Fe-N-C catalyst on a Hitachi SU 70 microscope at a working voltage of 5 kV. X-ray diffraction (XRD) on the Rigaku Ultima IV diffractometer using Cu Kα X-rays was used to identify the crystalline phases of each sample. X-ray photoelectron spectroscopy (XPS) was performed using a Kratos AXIS Ultra DLD XPS system equipped with a hemispherical energy analyzer and a monochromatic Al Kα source. The monochromatic Al Kα source operated at 15 keV and 150 W; For high-resolution scanning, the pass energy was fixed at 40 eV. All samples were prepared as pressed powder supported on metal rods for XPS measurement. The N_2 isotherm adsorption/desorption was recorded on the Micromeritics TriStar II at 77 K. Before the N_2 physical adsorption measurement, the sample was degassed under vacuum at 150 °C for 5 h. In addition, transmission electron microscopy (TEM), high resolution

TEM (HRTEM), high-angle circular dark field scanning TEM (HAADF-STEM), and electron energy loss spectrometer (EELS) were performed. A Renishaw Raman system was used to perform Raman spectroscopy under excitation at 514 nm. The sample was prepared as a powder on a standard microscope slide, and the excitation laser was focused through a 100× microscope objective to obtain a total interrogation spot size of 1.0 micrometer in diameter. The excitation power of all samples was kept constant at 150 μW. The Fe content of Fe-N-C-x was measured by inductively coupled plasma atomic emission spectroscopy (ICP-OES) (iCAP6300).

Electrochemical measurements

The electrochemical test was performed in a typical three-electrode system (CHI760e, Shanghai Chenhua Co., China) at room temperature. SCE (filled with saturation KCl solution) was used as reference electrode, graphite rod (6 mm in diameter) was used as counter electrode, and glassy carbon electrode (5 mm in diameter) was used as the working electrode. All potentials were converted according to E (RHE) = E (SCE) + (0.0591×pH + 0.2438) V. To prepare the catalyst ink, 5 mg of the prepared catalyst was dispersed in 490 μL of ethanol, 490 μL of deionized water and 20 μL of 5 wt.% Nafion. The mixture was sonicated for 60 min to form a homogeneous ink. The 10 μL ink was dropped on the glassy carbon electrode and then naturally dried to form a thin film, resulting in the catalyst loading of 0.255 mg cm^{-2} in 0.1 M KOH/0.1 M $HClO_4$. For comparison, 20 wt. % Pt/C at loading of 0.128 mg cm^{-2}, was prepared in the same way in 0.1 M KOH, 20 wt. % Pt/C at loading of 0.255 mg cm^{-2}, was prepared in the same way in 0.1 M $HClO_4$. All the tests were conducted in 0.1 M KOH/0.1 M $HClO_4$ solutions and O_2 or N_2 was ventilated at least 0.5 h to ensure the saturation of electrolyte before the test. The cyclic voltammogram (CV) curves under N_2 and O_2 saturation were tested at a scan rate of 10 mV s^{-1}. The linear sweep voltammogram (LSV) tests were recorded at the speed range of 400-1600 rpm at a scan rate of 10 mV s^{-1}. The Koutecky-Levich (K–L) plots were attained from polarization curves recorded at various potentials. The *n* value was estimated by the following equations:

$$\frac{1}{J} = \frac{1}{J_L} + \frac{1}{J_K} += \frac{1}{B\omega^{\frac{1}{2}}} + \frac{1}{J_K}$$

$$B = 0.62nFC_{O_2}(D_{O_2})^{\frac{2}{3}}\nu^{-\frac{1}{6}}$$

$$J_K = \frac{J \times J_L}{J_L - J}$$

Where F is the Faraday constant (96485 C mol^{-1}), C_{O_2} is the bulk concentration of O_2 (1.15×10^{-6} mol cm^{-3} in 0.1 M KOH), D_{O_2} is the diffusion coefficient of O_2 (1.90×10^{-5} cm^2 s^{-1}), and ν is the kinematic viscosity of the electrolyte (0.01 cm^2 s^{-1}).

The RRDE tests had the same voltage scanning range with CV at a scanning speed of 10 mV s^{-1} at electrode rotation speed of 1600 rpm, with the ring potential at 0.6 V (vs. RHE). The *n* value and H_2O_2 % were determined from the below equations:

$$n = \frac{4 \times I_D}{\frac{I_R}{N} + I_D}$$

$$H_2O_2(\%) = \frac{200 \times \frac{I_R}{N}}{\frac{I_R}{N} + I_D}$$

Where I_D is disk current, I_R is ring current. The current collection efficiency N for the Pt ring is 0.37.

Computational methods

All the density functional theory (DFT) calculations were performed using Vienna ab initio simulation package (VASP). The generalized gradient approximation (GGA) with the Perdew-Burke-Ernzerh of exchange correlation functional within the projector augmented wave method was utilized to model the electron-ion interaction. An energy cutoff of 500 eV for the plane-wave basis set was used. The convergence threshold was set to 10^{-6} eV in energy and 0.05 eV/Å in force, respectively. To prevent the interaction between two neighboring images, the vacuum layer thickness was set to 20 Å. A semi-empirical van der Waals (vdW)correction proposed by Grimme (DFT-D3) was included to account for the dispersion interactions. The free energy of each elementary step in the proton coupled electron transfer reactions was computed using the computational hydrogen electrode (CHE) model for oxygen reduction reaction (ORR).

Considering the O_2 molecular is not broken before reduction, the associative $4e^-$ reduction pathway was evaluated to be most feasible for ORR in this work, as follows:

$O_2 + * \rightarrow O_2*$

$O_2* + H_2O + e^- \rightarrow OOH* + OH^-$

$OOH* + e^- \rightarrow O* + OH^-$

$O* + H_2O + e^- \rightarrow OH* + OH^-$

$OH* + e^- \rightarrow OH^- + *$

where * represents the active site on the corresponding surface. The free energy of the reactants and each intermediate state at an applied electrode potential U are computed by the equation $\Delta G = \Delta E + \Delta ZPE - T\Delta S - neU$, where n is the electron number of such state and the ΔE was defined to be the difference of total energy of systems; ΔZPE and TΔS stand for the zero-point energy change and entropy change which were determined by employing the computed vibrational frequencies and standard tables for the reactants and products in the gas phase. To avoid the poorly description of triplet state of O_2 in DFT calculations, the free energy of $O_2(g)$ was evaluated to be $G_{O2} = 2G_{H2O} - 2G_{H2} - 4.92$ eV. The theoretical reaction overpotential (η_{ORR}) was evaluated to the difference between the minimum voltage needed for the ORR (1.23 V) and the voltage required for changing all the free-energy steps into downhill, which reads as:

$\eta_{ORR} = 1.23 - \min(\Delta G_{1\text{-}4})$

where min ($\Delta G_{1\text{-}4}$) is the step with the smallest ΔG value in $\Delta G_{1\text{-}4}$

Rechargeable Zn-air battery assembly and measurements

The home-made Zn-air batteries (ZABs) were assembled using polished zinc foil as the anode and aqueous solution containing 6 M KOH+0.2 M zinc acetate as the electrolyte. Fe-N-C-2 or commercial Pt/C +RuO_2 (with a mass ration of 1:1) coated on carbon paper was used as the air cathode. The total catalyst loading amount on carbon paper was 2.0 mg cm^{-2} for both Fe-N-C-2 and commercial Pt/C+RuO_2. Open circuit voltage and power density were measured using CHI 760E electrochemical workstation. For cycling stability test, the ZABs based on Fe-N-C-2 and Pt/C+RuO_2 were also assembled. The galvanostatic discharge/charge cycling stability for the ZABs was performed using a Neware battery testing station system (CT-4008T) with a cycling

interval of 20 min (10 min for discharging and 10 min for charging) under ambient condition.

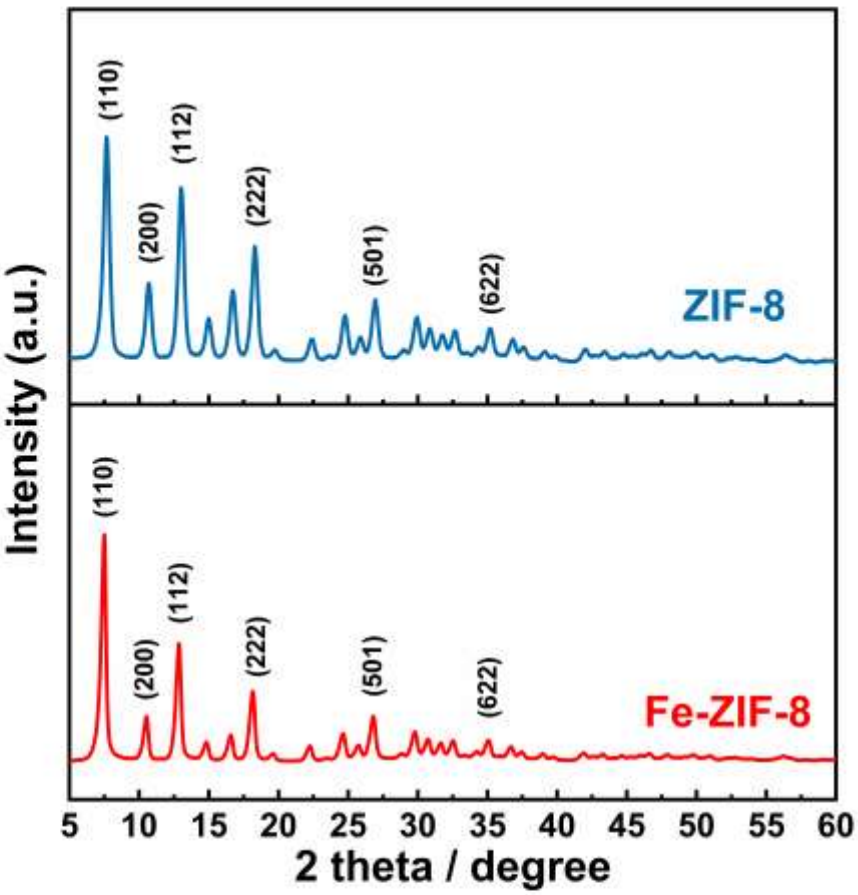

Figure S1. XRD patterns of ZIF-8 and Fe-ZIF-8.

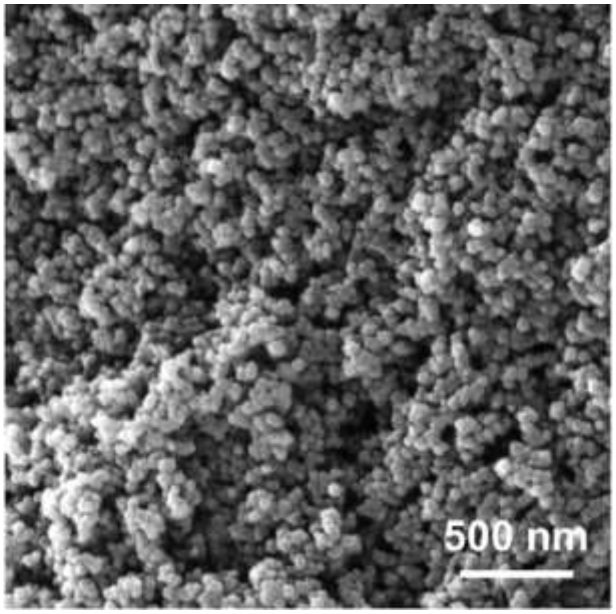

Figure S2. SEM image of the Fe-N-C-2 catalyst.

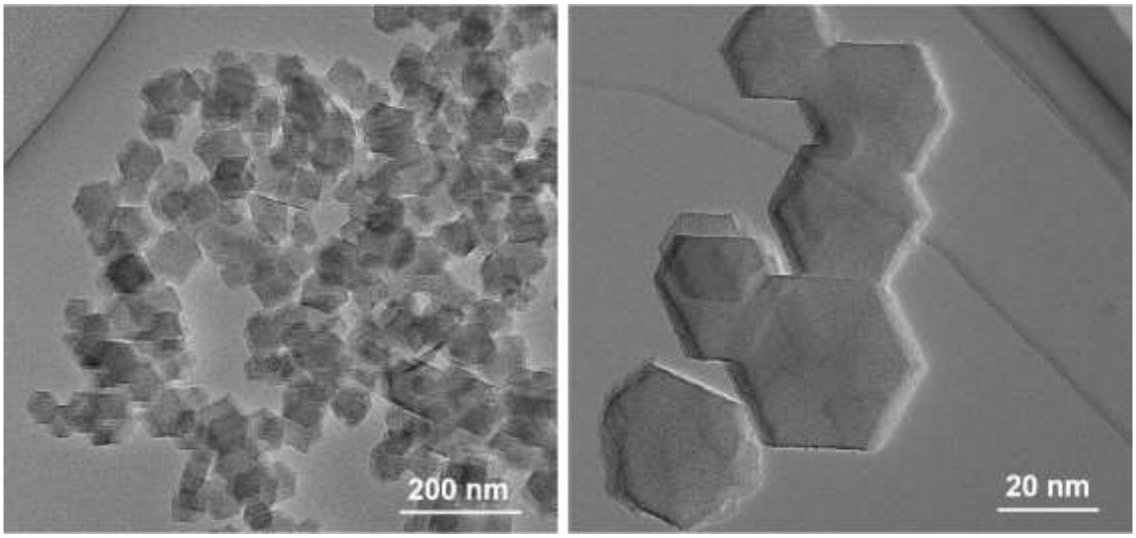

Figure S3. TEM images of Fe-N-C-0.

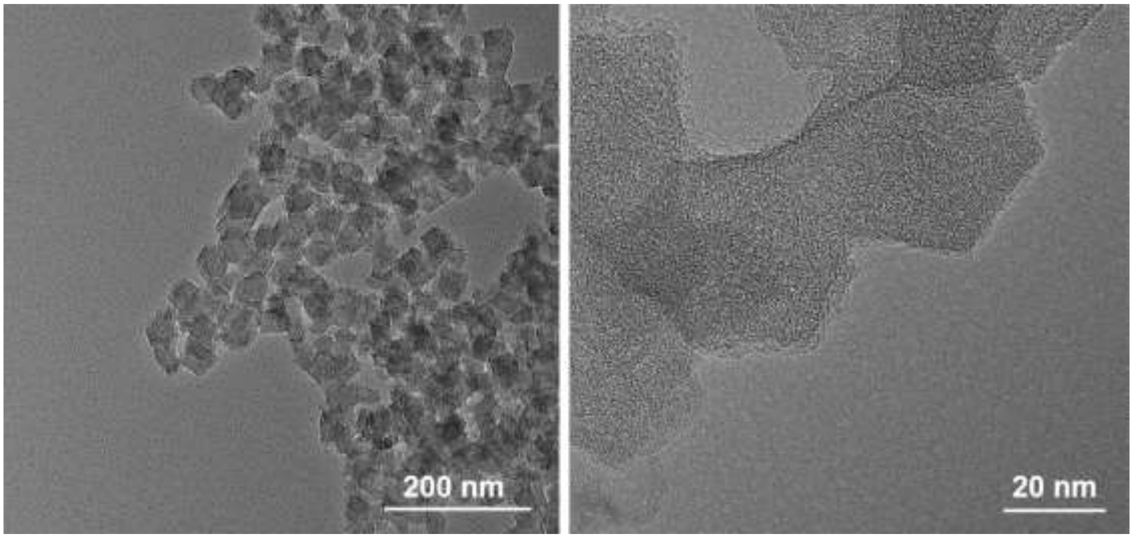

Figure S4. TEM images of Fe-N-C-1.

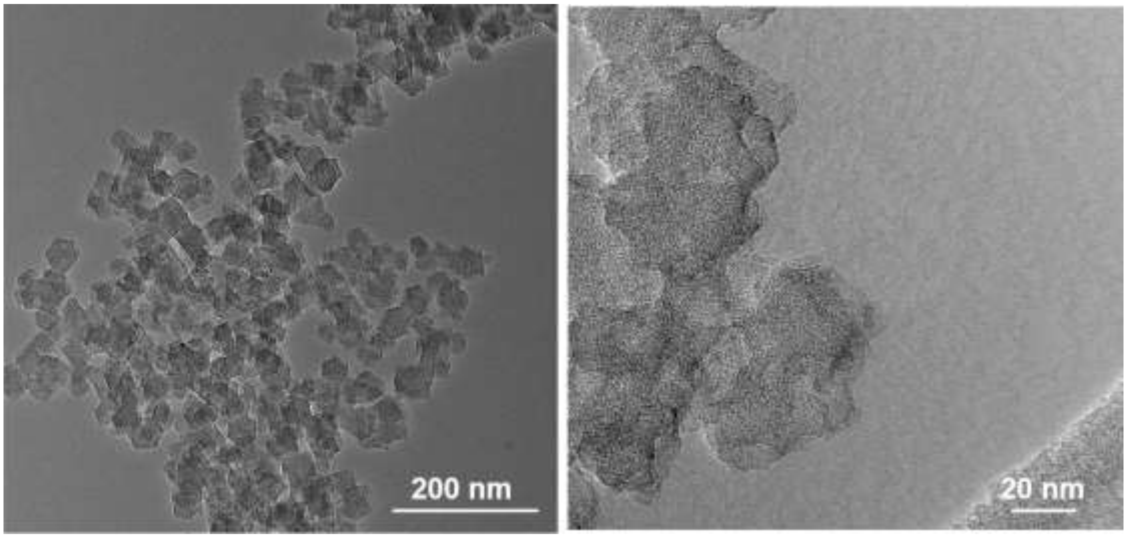

Figure S5. TEM images of Fe-N-C-3.

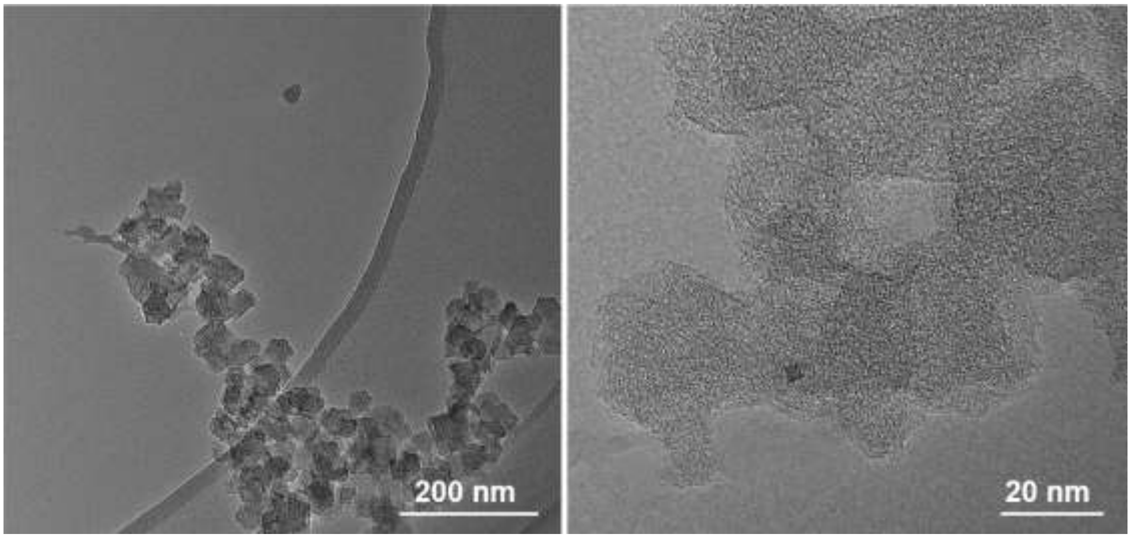

Figure S**6.** TEM images of Fe-N-C-4.

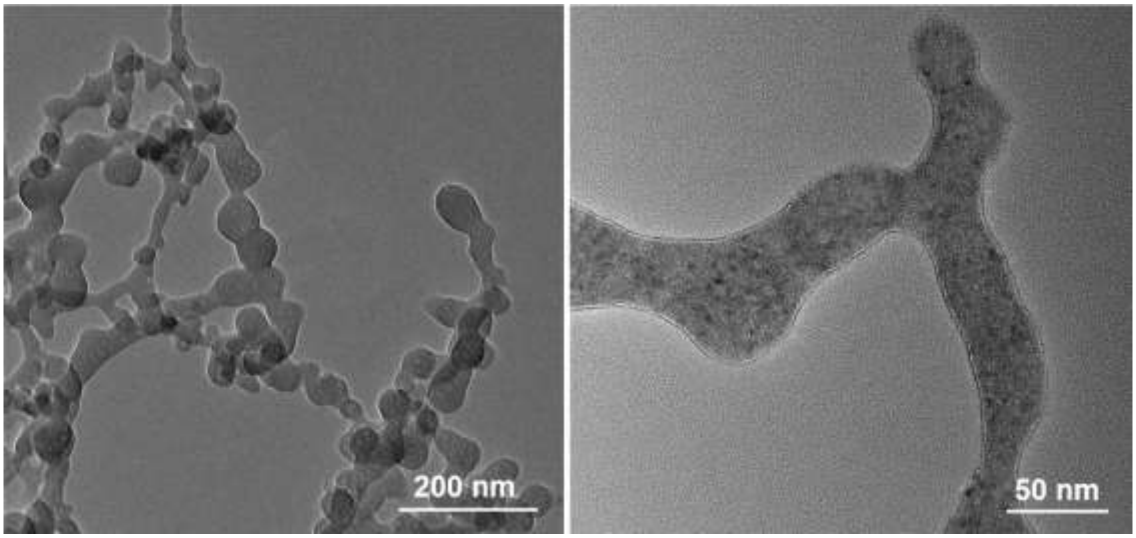

Figure S7. TEM images of Fe-N-C-5.

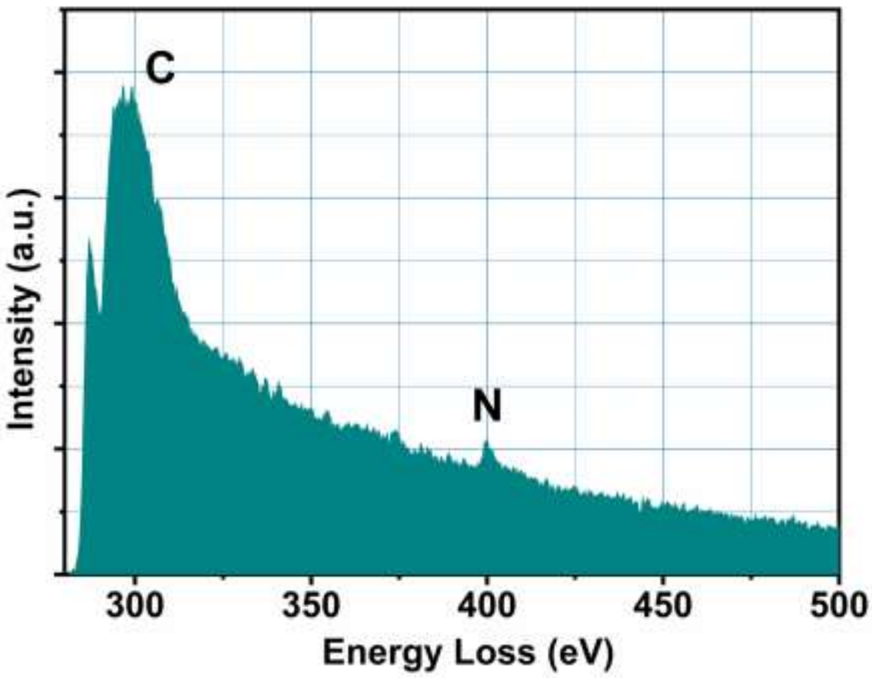

Figure S8. EELS analysis of selected paired spots of Fe-N-C-0 in HAADF-STEM images.

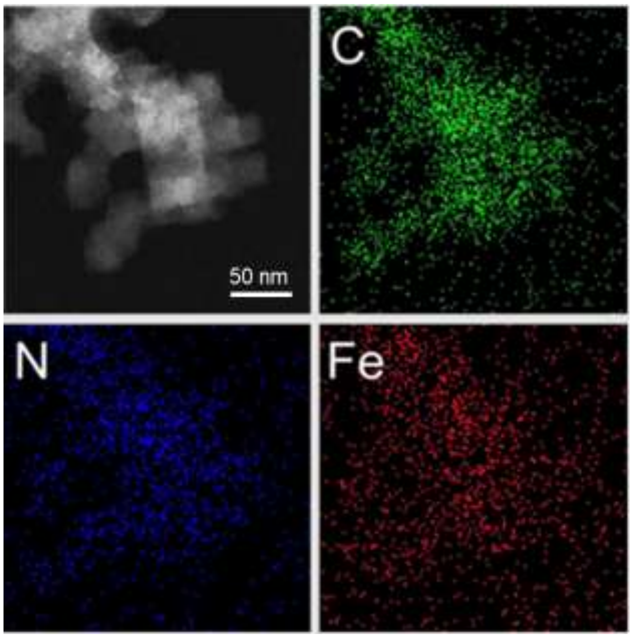

Figure S9. EDS of the Fe-N-C-2 catalyst.

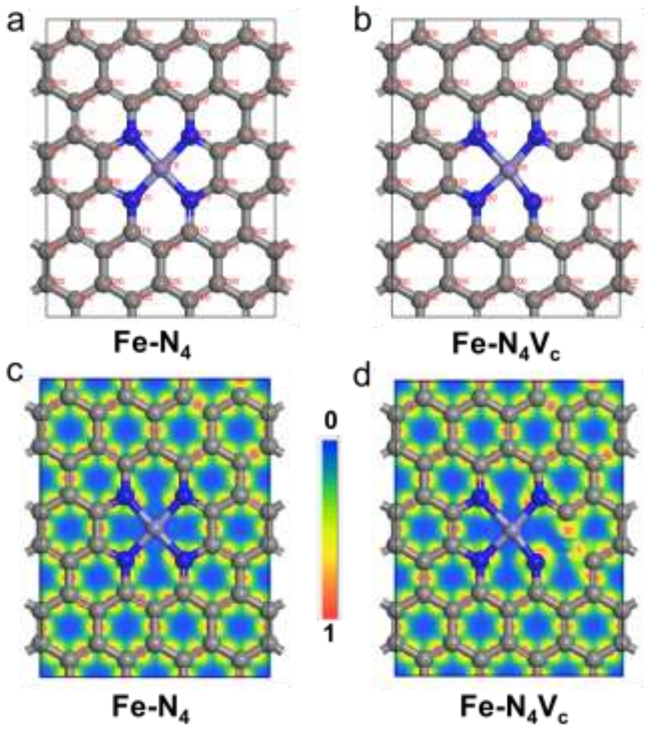

Figure S10. (a-b) Calculate the number of charges of Fe-N_4 and Fe-N_4Vc. (c-d) Computed charge distribution through ELF analysis of Fe-N_4 and Fe-N_4Vc.

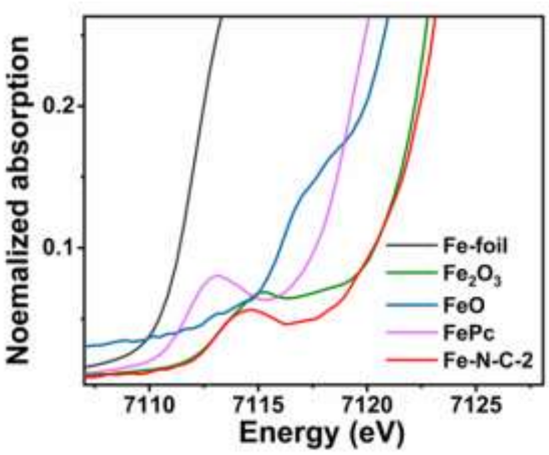

Figure S11. The Fe K-edge XANES spectra of Fe-N-C-2 and the references (Fe foil, FeO, Fe_2O_3 and FePc).

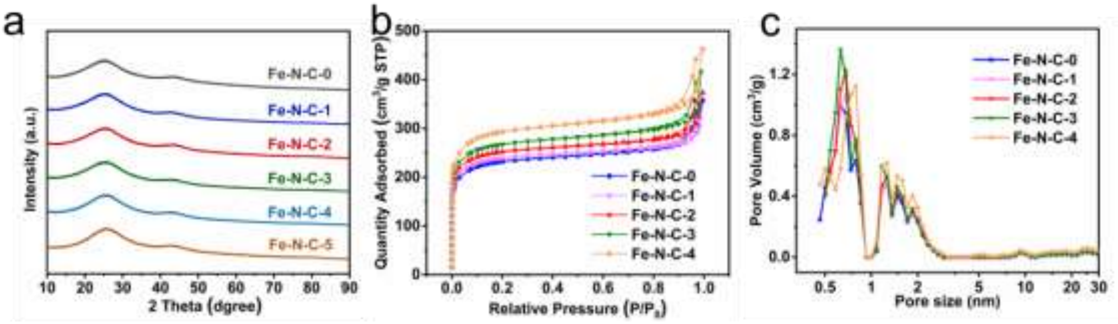

Figure S12. (a) PXRD patterns of Fe-N-C-*x*. (b) N_2 sorption isotherms of Fe-N-C-*x*. (c) The pore size distribution of Fe-N-C-*x*.

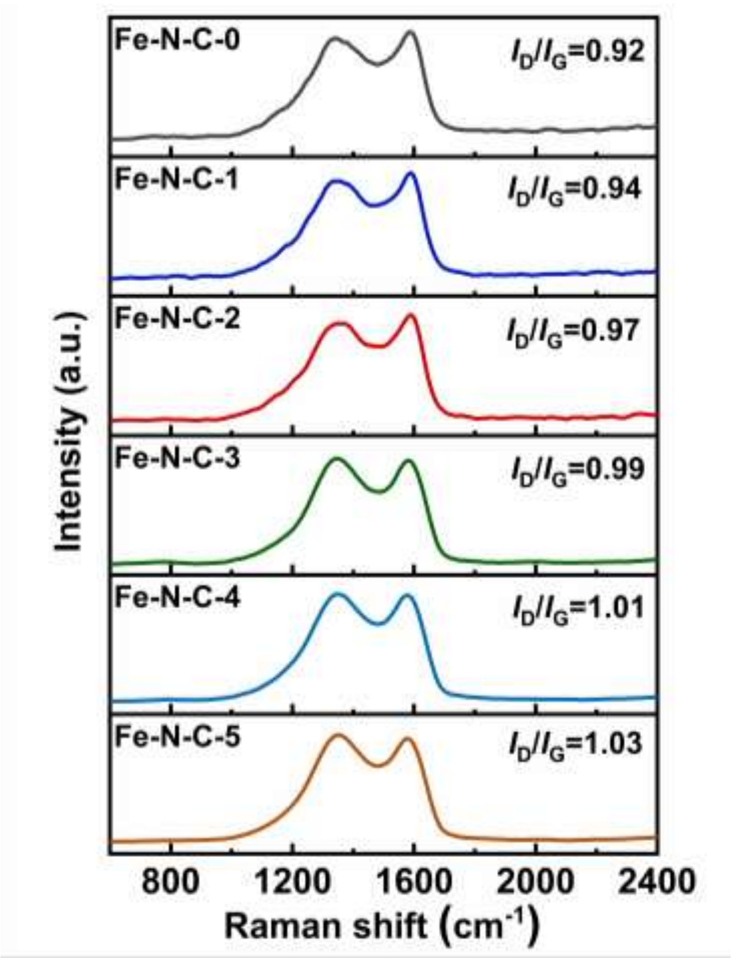

Figure S13. Raman spectra of Fe-N-C-*x*.

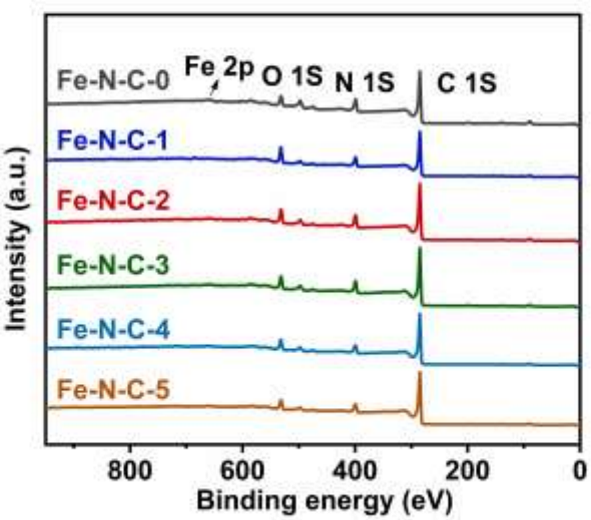

Figure S14. X-ray photoelectron spectroscopy (XPS) patterns of Fe-N-C-*x*.

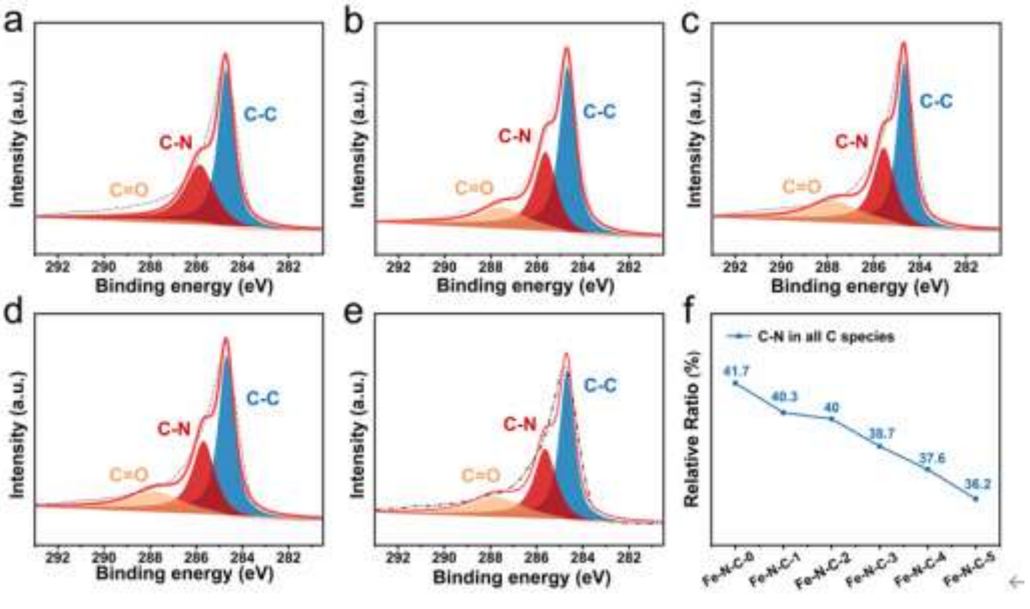

Figure S15. C *1s* XPS spectra of Fe-N-C-*x*. (a) Fe-N-C-0, (b) Fe-N-C-1, (c) Fe-N-C-3, (d) Fe-N-C-4 and (e) Fe-N-C-5. (f) The relative content ratio of C-N bonds in all C species (C-N, C-C, and C=O) of the six catalysts.

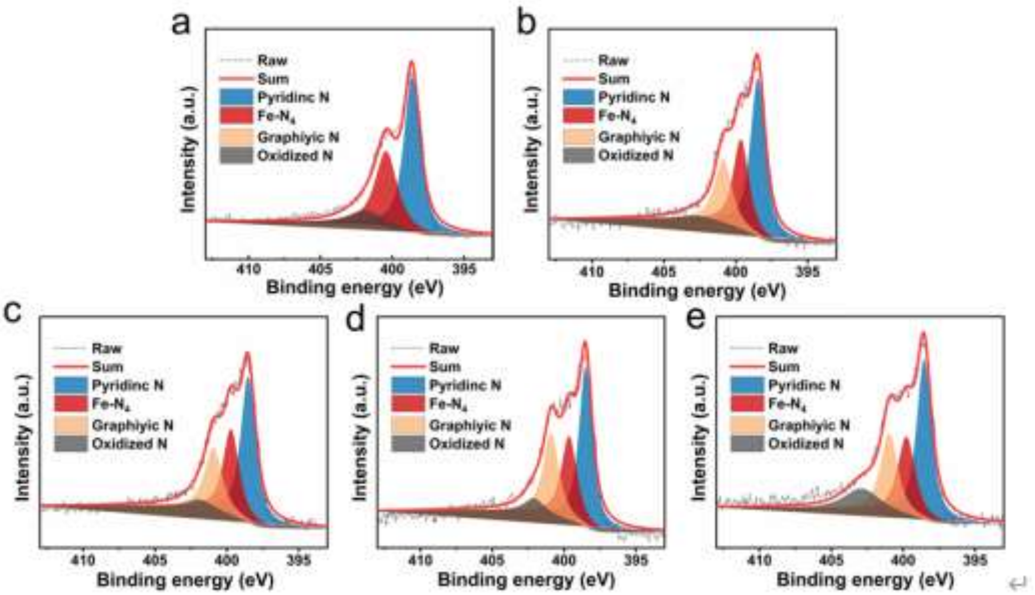

Figure S16. N *1s* XPS spectra of Fe-N-C-*x*. (a) Fe-N-C-0, (b) Fe-N-C-1, (c) Fe-N-C-3, (d) Fe-N-C-4 and (e) Fe-N-C-5.

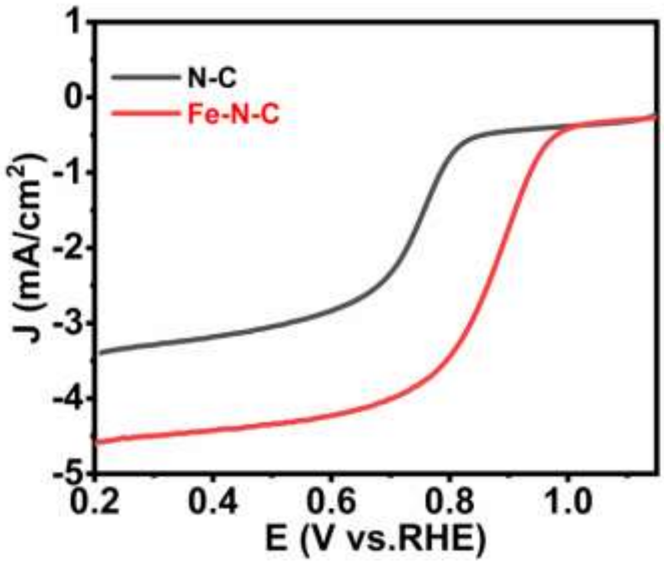

Figure S17. LSV curves of N-C and Fe-N-C in 0.1 M $HClO_4$ saturated with O_2 at a speed of 1600 rpm.

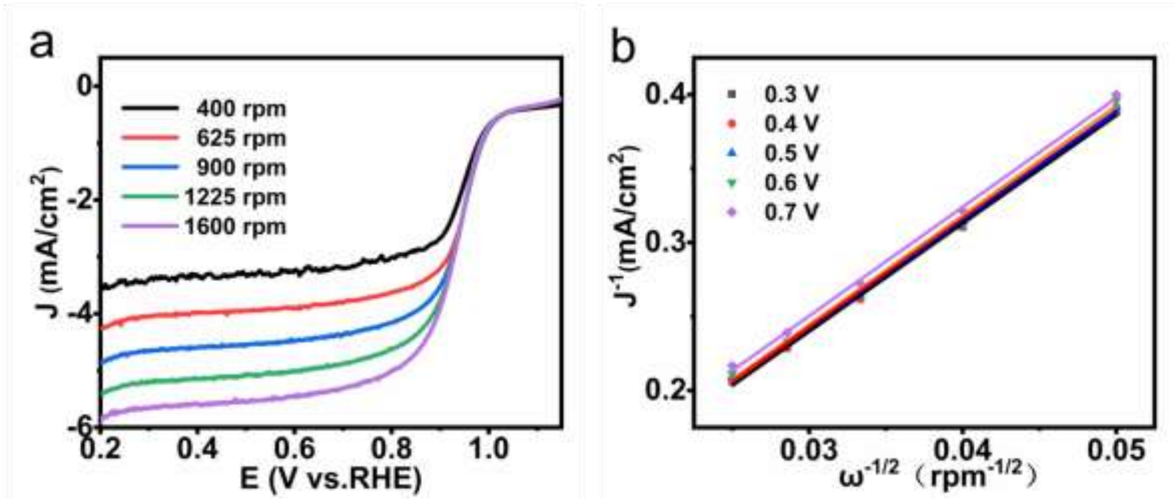

Figure S18. (a) **V**oltammograms of Fe-N-C-2 at various rotation rates, (b) the corresponding K-L plots.

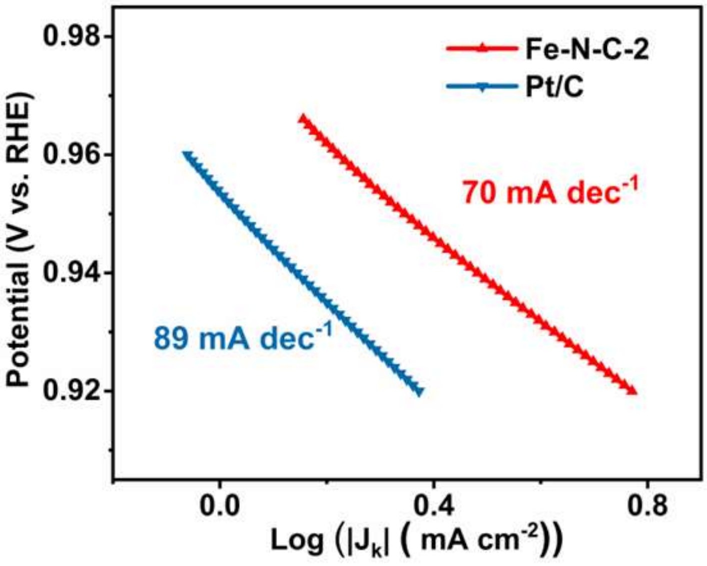

Figure S19. Tafel plots of Fe-N-C-2 and Pt/C in 0.1 M $HClO_4$.

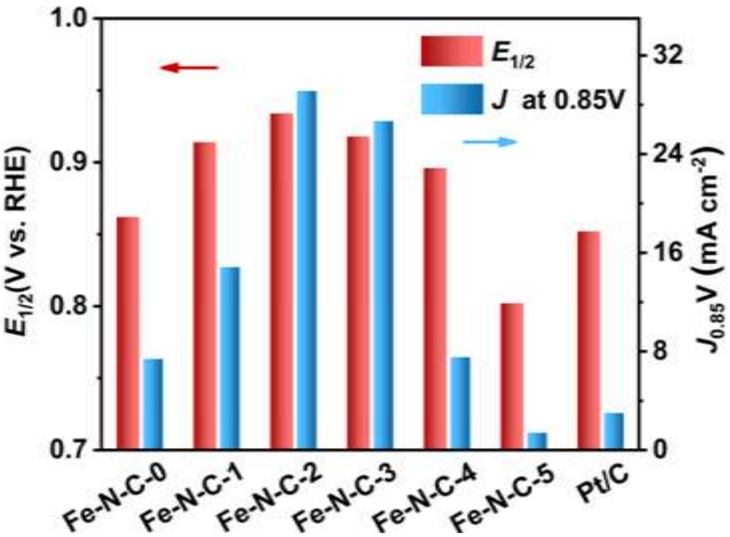

Figure S20. Kinetic current density *(J_k)* at 0.85 V and half-wave potential ($E_{1/2}$) for these catalysts.

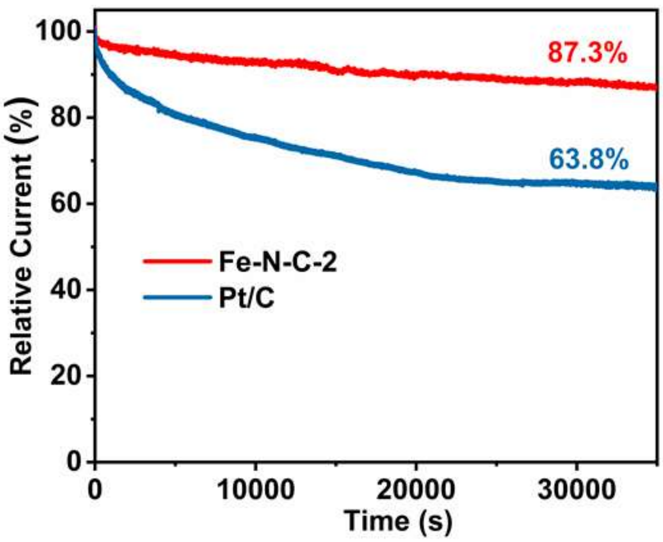

Figure S21. Chronoamperometric responses of Fe-N-C-2 and Pt/C at 900 rpm for 35000 s in 0.1 M $HClO_4$.

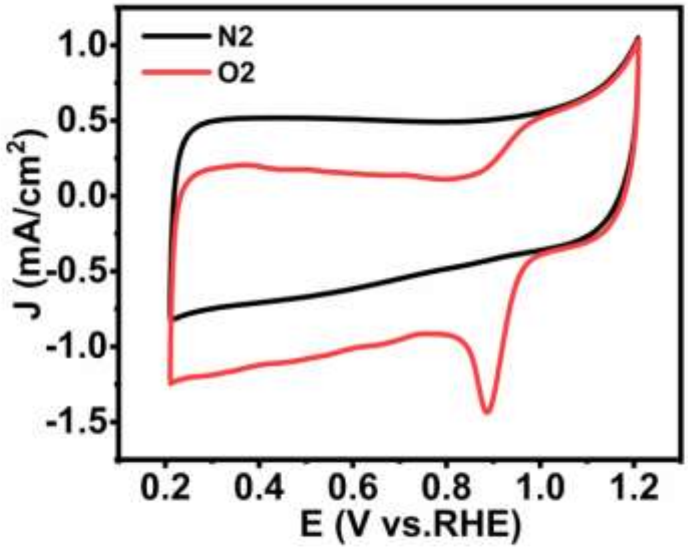

Figure S22. CV curves of Fe-N-C-2 catalyst in N_2 saturated solution and O_2 saturated solution in 0.1 M KOH.

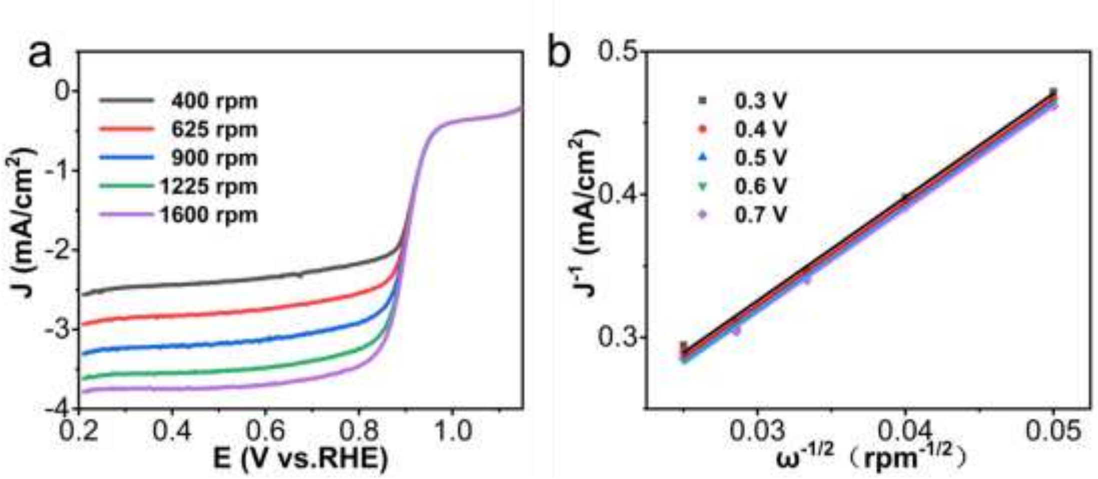

Figure S23. (a) **V**oltammograms of Fe-N-C-2 at various rotation rates, (b) the corresponding K-L plots.

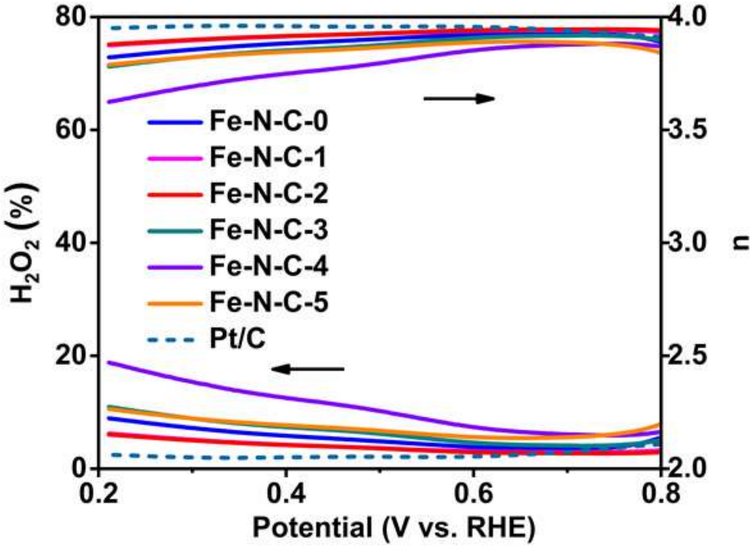

Figure S24. H_2O_2 yield and electron transfer number of Fe-N-C-*x* and Pt/C versus potential in 0.1 M KOH.

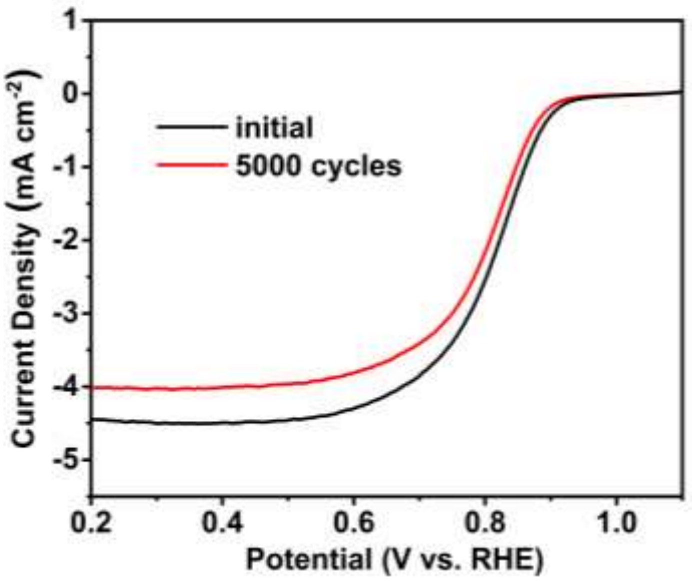

Figure S25. Stability test of Fe-N-C-0 in 0.1 M KOH.

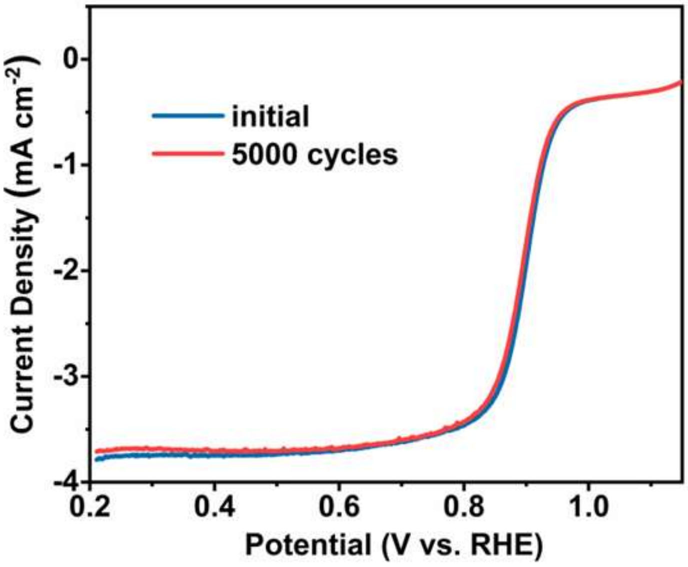

Figure S26. Stability test of Fe-N-C-2 in 0.1 M KOH.

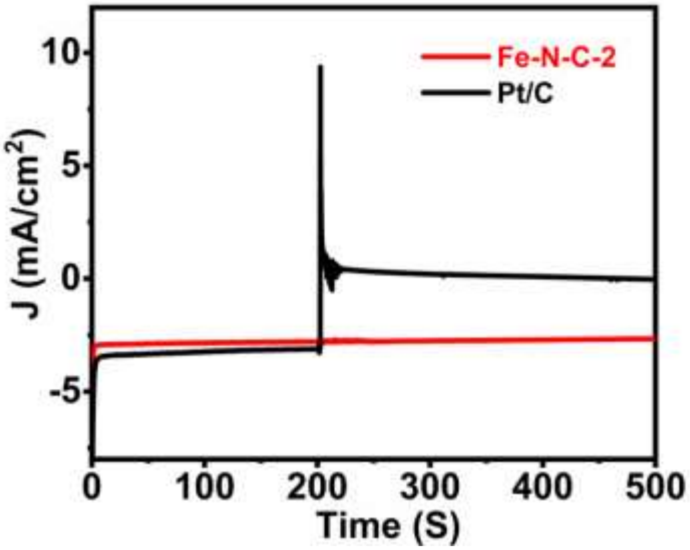

Figure S27. Current-time chronoamperometric response of Fe-N-C-2 and Pt/C with a rotating speed of 1600 rpm with addition of 3 M methanol in 0.1 M KOH.

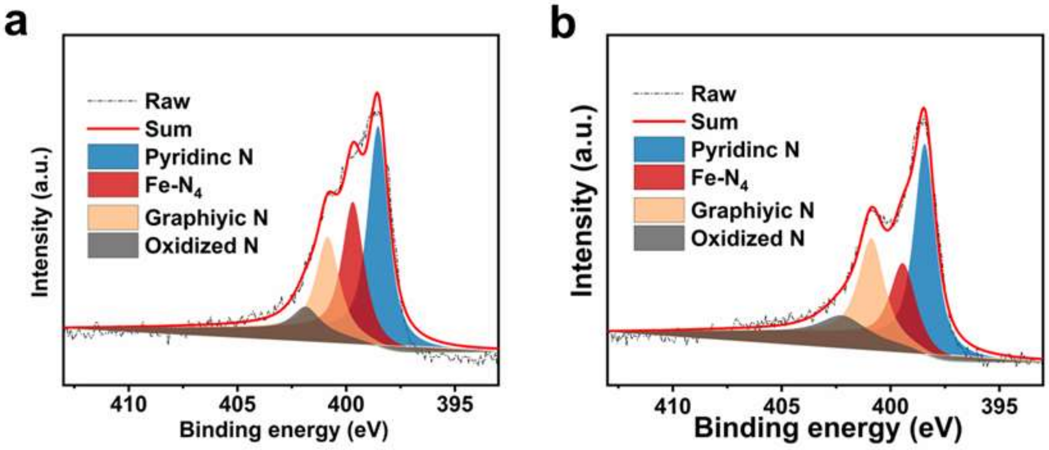

Figure S28. a) XPS spectra of N 1s in Fe-N-C-2 before 5000 CV cycles. b) XPS spectra of N 1s in Fe-N-C-2 after 5000 CV cycles.

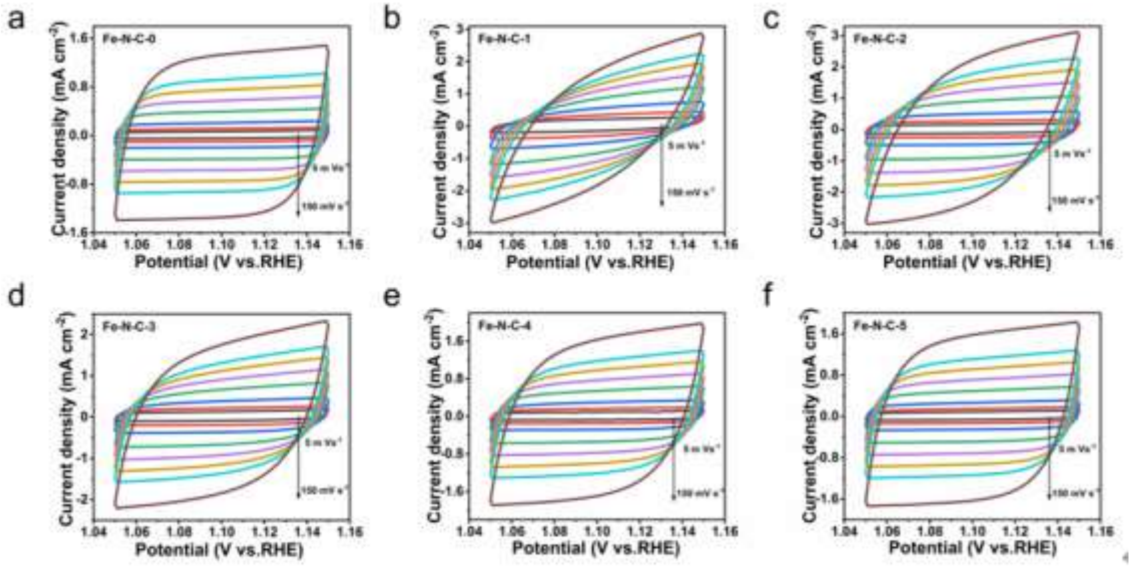

Figure S29. CV curves measured at a non-Faraday potential of 1.05 V to 1.15 V, with different scan rates. (a) Fe-N-C-0, (b) Fe-N-C-1, (c) Fe-N-C-2, (d) Fe-N-C-3, (e) Fe-N-C-4 and (f) Fe-N-C-5 in 0.1 M $HClO_4$.

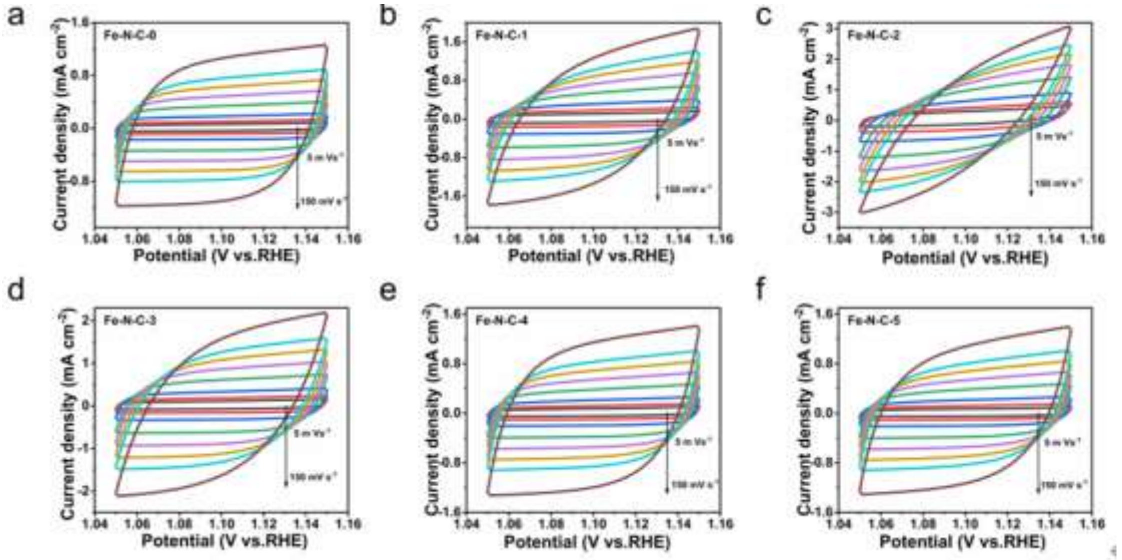

Figure S30. CV curves measured at a non-Faraday potential of 1.05 V to 1.15 V, with different scan rates. (a) Fe-N-C-0, (b) Fe-N-C-1, (c) Fe-N-C-2, (d) Fe-N-C-3, (e) Fe-N-C-4 and (f) Fe-N-C-5 in 0.1 M KOH.

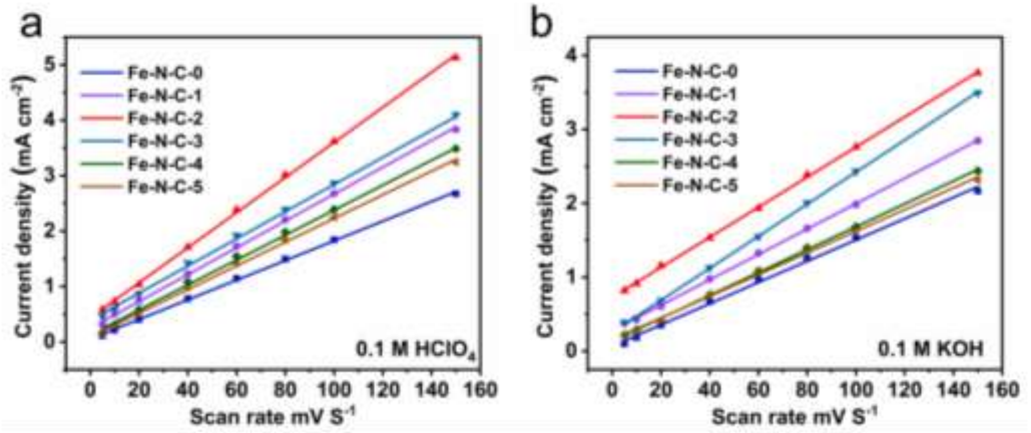

Figure S31. The corresponding double layer capacitance (C_{dl}) plots.

(a) Fe-N-C-*x* in 0.1 M $HClO_4$. (b) Fe-N-C-*x* in 0.1 M KOH.

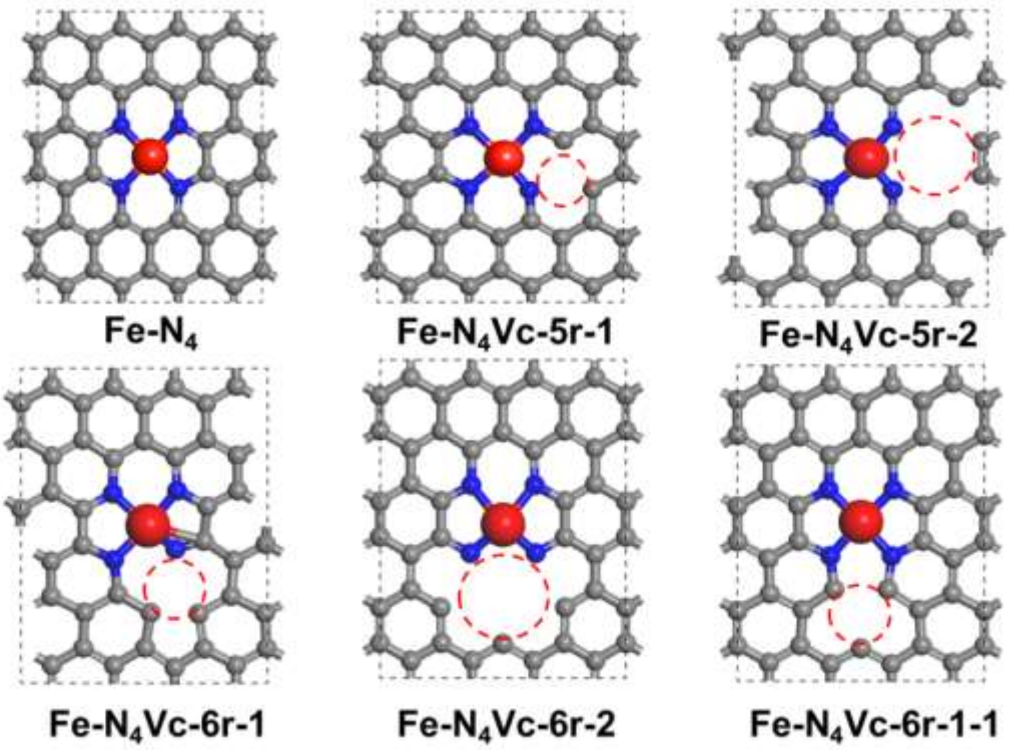

Figure S32. Five possible atomic configurations with different cracking degree (6r or 5r refers to the six- or five-membered Fe-N heterocyclic ring, respectively; 1 or 2 refers to the cleavage of one or two C-N bond adjacent to Fe-N_4, respectively).

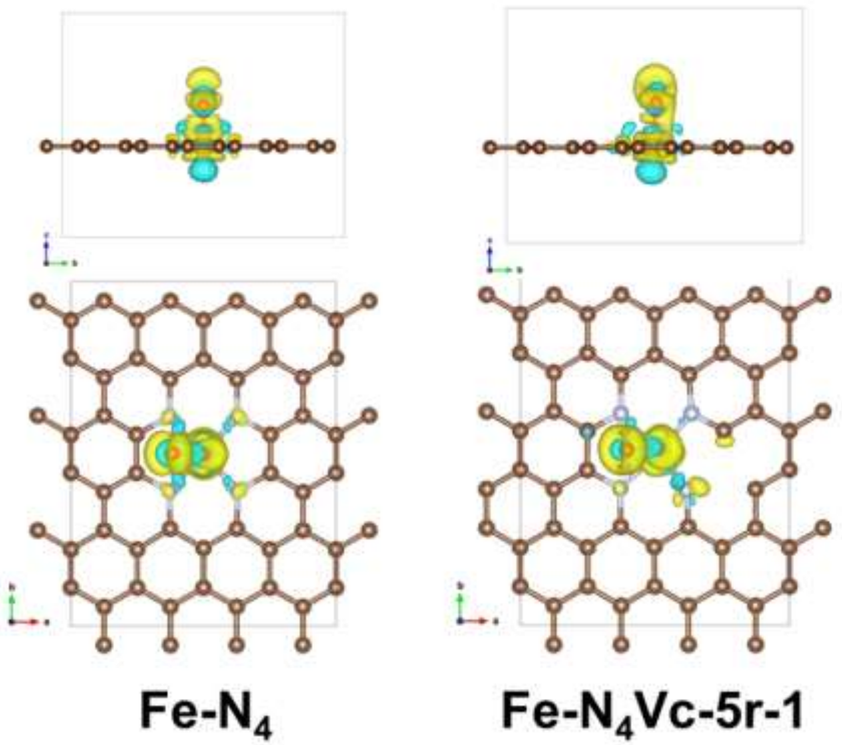

Figure S33. Calculated differential charge densities of Fe-N_4 and Fe-N_4Vc-5r-1 after binding to O_2 from top and side views. Yellow and blue bubbles represent electron and hole charges, respectively.

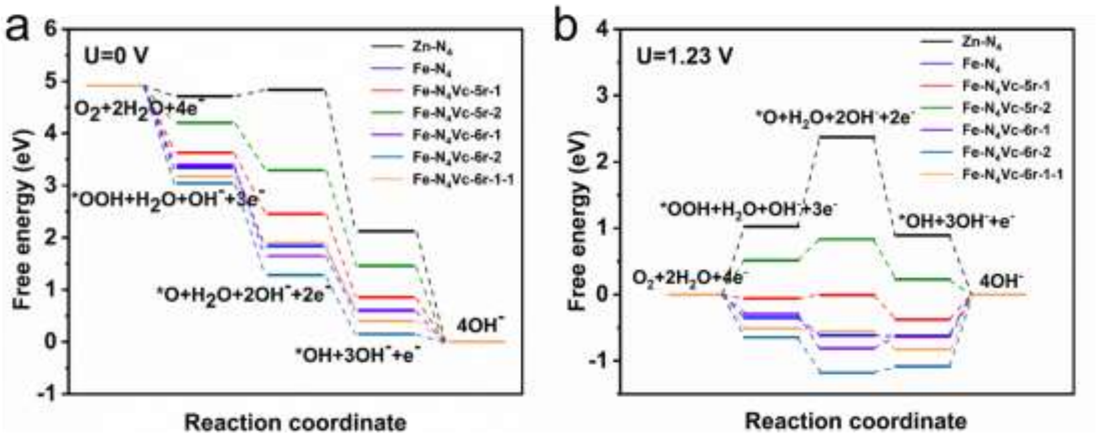

Figure S34. a) and b) ORR free energy diagrams in all structures at U = 0 and U =1.23 V.

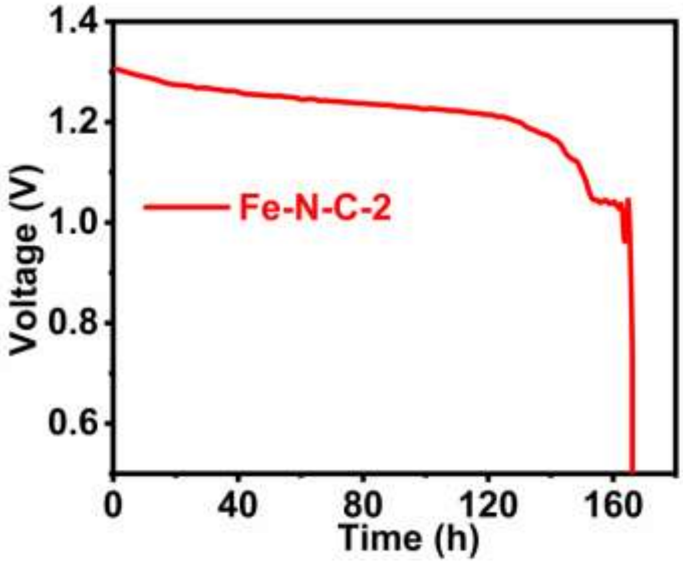

Figure S35. Long-term discharging performance of primary Zn-air battery with Fe-N-C-2 air cathode, respectively.

Table S1. Element contents determined by ICP.

Catalyst materials	Fe-N-C-0	Fe-N-C-1	Fe-N-C-2	Fe-N-C-3	Fe-N-C-4	Fe-N-C-5
Fe (wt. %)	3.1	3.6	4.5	5.3	6.4	7.1

Table S2. Element contents determined by XPS.

Catalyst materials	C (at. %)	N (at. %)	O (at. %)	Zn (at. %)	Fe (at. %)
Fe-N-C-0	81.7	10.19	5.56	2.23	0.31
Fe-N-C-1	75.53	11.28	10.75	1.99	0.45
Fe-N-C-2	76.83	12.45	7.99	2.14	0.59
Fe-N-C-3	77.6	12.26	7.56	1.98	0.6
Fe-N-C-4	80.68	9.96	6.86	2.08	0.68
Fe-N-C-5	81.49	9.64	6.3	1.82	0.75

Table S3. Comparison samples of the as-prepared Fe-N-C-*x* electrocatalysts in 0.1 M $HClO_4$.

catalysts	Onset Potential (V)	$E_{1/2}$ (V vs.RHE)	$J_{Limiting}$ (mA cm^{-2})
Fe-N-C-0	1.003	0.862	4.611
Fe-N-C-1	1.023	0.914	5.033
Fe-N-C-2	1.105	0.934	5.821
Fe-N-C-3	1.017	0.918	5.025
Fe-N-C-4	0.995	0.896	5.053
Fe-N-C-5	0.909	0.802	6.056

Table S4. Comparison samples of the as-prepared Fe-N-C-*x* electrocatalysts in 0.1 M KOH.

catalysts	Onset Potential (V)	$E_{1/2}$ (V vs.RHE)	$J_{Limiting}$ (mA cm^{-2})
Fe-N-C-0	0.962	0.803	4.183
Fe-N-C-1	0.993	0.892	3.531
Fe-N-C-2	1.015	0.901	4.596
Fe-N-C-3	0.992	0.895	4.581
Fe-N-C-4	0.984	0.885	3.252
Fe-N-C-5	0.975	0.871	3.706

Table S5. Element contents of Fe-N-C-2 before and after 5000 CV cycles determined by XPS.

Catalyst materials	C (at. %)	N (at. %)	O (at. %)	Zn (at. %)	Fe (at. %)
Fe-N-C-2 (before)	76.83	12.45	7.99	2.14	0.59
Fe-N-C-2 (after)	79.1	12.14	6.37	1.99	0.51

Table S6. Summary of previously reported single-atomic catalysts in terms of their ORR performance.

catalysts	$E_{1/2}$/V (vs.RHE) in 0.1 M KOH	$E_{1/2}$/V(vs.RHE) in 0.1 M $HClO_4$	Power density / mW cm^{-2}	Ref.
Fe-N-C-2	0.901	0.934	218	This work
Cu@Fe-N-C	0.892	0.761	92	*Adv. Funct. Mater.* **2018**, *28*, 1802596[1]
FePc $(CN)_8$	0.910	0.801	--	ACS Catal. **2019**, 9, 6252-6261[2]
Fe-N-C/H_2O_2	0.92	0.79	--	Appl. Catal. B 263 (**2020**) 118347[3]
TimB-Fe_5-C	0.89	0.78	--	*Nano Energy* **2020**.105533[4]
Co_2/FeN@CHC	0.915	0.812	232.4	*Adv. Mater.* **2021**, 2104718[5]
SACe-N/PC	0.88	0.75	155	Nano Lett. **2021**, 21, 4508-4515[6]
Fe, Mn/N-C	0.928	0.804	160.8	NATURE COMMUNICATIONS (**2021**) 12:1734[7]
Pt/C	0.863	0.852	149	

References

[1] Z. Wang, H. Jin, T. Meng, K. Liao, W. Meng, J. Yang, D. He, Y. Xiong, S. Mu, *Adv. Funct. Mater.* **2018**, *28*, 1802596.

[2] Y. Wang, M. Wang, Z. Zhang, Q. Wang, Z. Jiang, M. Lucero, X. Zhang, X. Li, M. Gu, Z. Feng, Y. Liang, *ACS Catal.* **2019**, *9*, 6252.

[3] X. Wei, X. Luo, H. Wang, W. Gu, W. Cai, Y. Lin, C. Zhu, *Appl. Catal. B.* **2020**, *263*, 118347.

[4] X. Lin, P. Peng, J. Guo, L. Xie, Y. Liu, Z. Xiang, *Nano Energy.* **2021**, *80*, 105533.

[5] Z. Wang, X. Jin, C. Zhu, Y. Liu, H. Tan, R. Ku, Y. Zhang, L. Zhou, Z. Liu, S. J. Hwang, H. J. Fan, *Adv Mater.* **2021**.
[6] J. C. Li, X. Qin, F. Xiao, C. Liang, M. Xu, Y. Meng, E. Sarnello, L. Fang, T. Li, S. Ding, Z. Lyu, S. Zhu, X. Pan, P. X. Hou, C. Liu, Y. Lin, M. Shao, *Nano Lett.* **2021**, *21*, 4508.
[7] G. Yang, J. Zhu, P. Yuan, Y. Hu, G. Qu, B. A. Lu, X. Xue, H. Yin, W. Cheng, J. Cheng, W. Xu, J. Li, J. Hu, S. Mu, J. N. Zhang, *Nat Commun.* **2021**, *12*, 1734.
